# Small Feature‐Size Transistors Based on Low‐Dimensional Materials: From Structure Design to Nanofabrication Techniques

**DOI:** 10.1002/advs.202400500

**Published:** 2024-06-17

**Authors:** Xiaqing Fu, Zhifang Liu, Huaipeng Wang, Dan Xie, Yilin Sun

**Affiliations:** ^1^ School of Microelectronics Shanghai University Shanghai 201800 P. R. China; ^2^ School of Integrated Circuits and Electronics Beijing Institute of Technology Beijing 100081 P. R. China; ^3^ School of Integrated Circuits Beijing National Research Center for Information Science and Technology (BNRist) Tsinghua University Beijing 100084 P. R. China

**Keywords:** nanofabrication, nano‐gate transistors, small feature‐size transistors, vertical transistors

## Abstract

For several decades after Moore's Law is proposed, there is a continuous effort to reduce the feature‐size of transistors. However, as the size of transistors continues to decrease, numerous challenges and obstacles including severe short channel effects (SCEs) are emerging. Recently, low‐dimensional materials have provided new opportunities for constructing small feature‐size transistors due to their superior electrical properties compared to silicon. Here, state‐of‐the‐art low‐dimensional materials‐based transistors with small feature‐sizes are reviewed. Different from other works that mainly focus on material characteristics of a specific device structure, the discussed topics are utilizing device structure design including vertical structure and nano‐gate structure, and nanofabrication techniques to achieve small feature‐sizes of transistors. A comprehensive summary of these small feature‐size transistors is presented by illustrating their operation mechanism, relevant fabrication processes, and corresponding performance parameters. Besides, the role of small feature‐size transistors based on low‐dimensional materials in further reducing the small footprint is also clarified and their cutting‐edge applications are highlighted. Finally, a comparison and analysis between state‐of‐art transistors is made, as well as a glimpse into the future research trajectory of low dimensional materials‐based small feature‐size transistors is briefly outlined.

## Graphical abstract

With the unique device structures and the development of 2D materials, many transistors with ultrasmall feature‐sizes are successfully fabricated. This review summarizes the novel, advanced methods for fabricating ultrasmall nanoscale transistors, and discusses the challenges and future trends of ultrasmall devices.

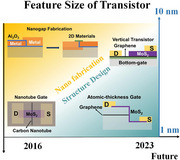

## 1 Introduction

Gordon Moore proposed Moore's law, which states that the computational capability and transistor density in a chip will double approximately every 2 years.^[^ ^1^ ^]^ Since 1965, it has consistently served as a dependable indicator of the rate at which electronic technology progresses. Consequently, it has stimulated the emergence of numerous groundbreaking technologies, while simultaneously enhancing the levels of integration.^[^ ^2^ ^]^ Referring to the former research and experiences, chips would go faster, consume less power,^[^ ^3^ ^]^ become cheaper, and improve their system reliability when they are scaled to a smaller size with little need for tradeoffs.^[^ ^4^ ^]^ During certain periods of scaling, there were major changes from Si‐based bipolar transistors to p‐channel metal‐oxide‐semiconductor (MOS) transistors, n‐channel MOS transistors, and finally to complementary MOS (CMOS) planar transistors in the 1980s, which have remained as the dominate technology for the past decades.^[^ ^2^ ^]^ However, when the size of the silicon device is scaled down to tens of nanometers, some challenges prevent researchers from further scaling the silicon device.

In specific, challenges arise when the channel length falls below the required minimum length of the silicon channel, causing the gate to lose control of the current. These phenomena are commonly known as short‐channel effects (SCEs).^[^ ^5^ ^]^ To be more precise, the majority of challenges arise from SCEs during the scaling process,^[^ ^6^ ^]^ when the channel length of a MOSFET reaches a point where it is nearly equal to the space charge regions of the source and drain junctions with the substrate.^[^ ^7^ ^]^ The SCEs mainly include: i) Direct source‐to‐drain tunneling current. ii) The loss of gate electrostatic control on the channel severely degrades the Off‐state leakage currents.^[^ ^8^ ^]^ iii) the drain‐induced barrier lowering (DIBL) effect.^[^ ^6^ ^]^ Besides SCEs, the resolution of lithography is also a critical limiting factor that necessitates the exploration of novel lithographic approaches to realize future generations of devices.^[^ ^9^ ^]^ For photolithography, since its resolution depends upon the wavelength of the light source,^[^ ^10^ ^]^ it is necessary to achieve short‐wavelength lithography like UV lithography or even extreme ultraviolet (EUV) lithography. However, UV lithography has not yet been put into industrial production on a large scale^[^ ^11^ ^]^ since it requires a highly‐level fabricated radiation source.^[^ ^12^ ^]^ There are also some advanced alternative lithography strategies, such as electron beam lithography (EBL), x‐ray lithography, and ion beam lithography.^[^ ^9^ ^]^ However, despite the ability to accelerate electron beams to high energies and focus them to achieve a theoretical lithograph spot size of 1 nm, it remains challenging to scale down the gate length below 5 nm. This limitation applies to both planar global (back) gate transistors and local (top) gate transistors using EBL.^[^ ^13^ ^]^

In order to closely follow Moore's law and further scale the devices to the sub‐10 nm region, limitations have to be overcome by either finding new materials^[^ ^14^, ^15^ ^]^or designing new device structures.^[^ ^16^, ^17^, ^18^ ^]^ From the perspective of materials, materials with heavier carrier effective mass, larger band gap, and lower in‐plane dielectric constant can yield lower direct source‐to‐drain tunneling currents,^[^ ^19^ ^]^ uniform and atomically thin semiconductors with low in‐plane dielectric constants are desirable for enhanced electrostatic control. Therefore, materials with the idealized properties mentioned above, such as transition metal dichalcogenides (TMDs), one kind of layered 2D material, show great potential in the field of highly scaled electronic devices.^[^ ^20^, ^21^, ^22^ ^]^ Besides TMDs, 2D graphene composed of single‐layer carbon atoms, is helpful in further scaling by being the material of electrodes (S/D electrodes, gate).^[^ ^23^, ^24^ ^]^ As S/D electrodes, the natural thickness of graphene film can still enable sufficient electrostatic control when the channel length is extremely scaled; as a gate electrode, graphene film has a high electrical conductivity that can minimize the voltage drop along the gate layer.^[^ ^6^ ^]^ Moreover, graphene exhibits a field‐tunable work‐function and partial electrostatic transparency,^[^ ^25^, ^26^ ^]^ It can therefore function as an “active” contact in tunable graphene‐semiconductor or graphene‐insulator junctions to enable new applications.^[^ ^27^, ^28^, ^29^, ^30^ ^]^ At last, finding high k material as gate insulators is also helpful in further scaling the device by reducing effective oxide thickness (EOT) without the need for greatly reducing the physical thickness of gate^[^ ^11^ ^]^ thus gaining lower gate tunnel currents.^[^ ^31^ ^]^

In addition to the excavation of new materials, the design of a new device structure is also crucial to trace after Moore's law, just as mentioned above. For example, FinFETs and their variants show great potential in scalability and manufacturability for nanoscale CMOS because there are little SCEs in FinFETs since gate control is enhanced by surrounding channels with gates.^[^ ^32^ ^]^ But when the gate length is scaled down to 10 nm, gate‐all‐around devices have been demonstrated to provide an even better gate control than FinFETs. However, the structures mentioned above still require highly leveled lithography^[^ ^33^ ^]^ (channel length is determined by the resolution of lithography). These complicated and expensive fabrication processes pose a key challenge for the development and commercialization of highly scaled devices. To overcome this limitation, for example, vertical transistors have been developed, where a thin‐film semiconductor body is simply sandwiched between the source and drain electrodes, and the channel length is determined by its body thickness.^[^ ^14^, ^24^ ^]^ Within this kind of structure, high‐resolution lithography is not necessarily needed in the corresponding fabrication flow.

**Figure 1:**
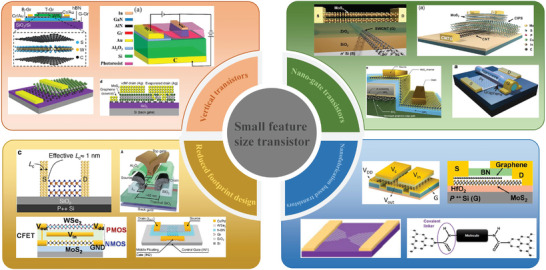
Overview of this review. The whole review is based on small feature‐size transistors including vertical field effect transistors, Reproduced with permission.^[^ ^47^ ^]^ Copyright 2022, American Chemical Society. Reproduced with permission.^[^ ^48^ ^]^ Copyright 2021, The Author(s), under exclusive license to Springer Nature Limited. Reproduced with permission.^[^ ^68^ ^]^ Copyright 2023, American Chemical Society. nano‐gate transistors, Reproduced with permission.^[^ ^71^ ^]^ Copyright 2016, American Association for the Advancement of Science. Reproduced with permission.^[^ ^77^ ^]^ Copyright 2020, Science China Press. Published by Elsevier B.V. and Science China Press. All rights reserved. Reproduced with permission.^[^ ^6^ ^]^ Copyright 2022, The Author(s), under exclusive license to Springer Nature Limited. Reproduced with permission.^[^ ^97^ ^]^ Copyright 2010, Springer Nature Limited. nanofabrication‐based transistors, Reproduced with permission.^[^ ^124^ ^]^ Copyright 2017, American Chemical Society. Reproduced with permission.^[^ ^126^ ^]^ Copyright 2017, American Chemical Society. Reproduced with permission.^[^ ^127^ ^]^ Copyright 2023, American Chemical Society. and designs for reduced footprint. Reproduced with permission.^[^ ^133^ ^]^ Copyright 2019, American Chemical Society. Reproduced with permission.^[^ ^134^ ^]^ Copyright 2017, The American Association for the Advancement of Science. Reproduced with permission.^[^ ^137^ ^]^ Copyright 2021, IEEE. Reproduced with permission.^[^ ^143^ ^]^ Copyright 2023, American Chemical Society.

Here, we reviewed the state‐of‐the‐art small feature‐size transistors based on low‐dimensional materials from the perspective of device structure design and nanofabrication techniques, separating it from the current review works on 2D material‐based transistors, which focused on the preparation techniques of 2D materials and transistor performance with specific structures.^[^ ^34^, ^35^, ^36^ ^]^ This review will introduce several advanced device structures, including vertical transistors and nano‐gate length transistors, and nanofabrication techniques toward small feature‐size transistors. Such new device structures and fabrication techniques will bring new ideas for the development of small feature‐size transistors and contribute to the further scaling down of their footprints. Finally, a short perspective on the challenges and future developments of high‐performance small feature‐size transistors is proposed. **Figure** **1** shows the overview of this review.

## 2 Vertical Transistor

Just as mentioned in the introduction part, since the channel of a vertical transistor is sandwiched between source and drain electrodes and its length is determined by body thickness, so vertical transistor is an alternative way to achieve ultra‐small channel length beyond the resolution limitation of lithography.^[^ ^24^ ^]^ What's more, with the development of material science, combining vertical structure with 2D materials offers interesting opportunities for digital and high‐frequency electronics.^[^ ^37^ ^]^ In this section, we are going to introduce 2D material based‐vertical transistors with three different operation mechanisms: tunneling field effect transistor,^[^ ^38^, ^39^, ^40^ ^]^ tunable Schottky barrier transistor,^[^ ^24^, ^41^ ^]^ and hot electron transistor.^[^ ^42^, ^43^, ^44^ ^]^ A review of these three types of transistors about their basic structure and device mechanisms will be made, as well as an introduction to several recently designed advanced transistors.

### 2.1 Vertical Tunneling Field Effect Transistor

**Figure 2:**
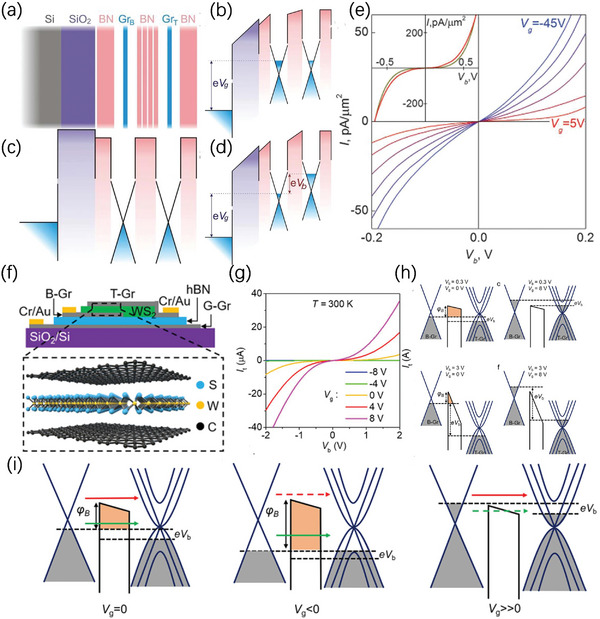
a) Layer stacking structure of the first vertical tunneling transistor, Si works as a back gate and SiO_2_ works as a gate dielectric. b) Under the effect of the back‐gate bias V_g_. c) Energy band diagrams under equilibrium conditions. d) Under the effect of V_g_ and a bias V_b_ between the two Gr layers. (e) *I*–*V*_s_ for different V_g_. Reproduced with permission.^[^ ^46^ ^]^ Copyright 2012, American Association for the Advancement of Science. f) The schematic of the graphene‐WS_2_‐graphene vertical transistor. g) The *I‐*‐V_b_ curves under different V_g_. h) The band diagram under small V_g_ (0.3 V) and large V_g_ (3 V) under different bias voltage. i) Energy band diagrams of GWG tunneling transistors under different V_g_ and a fixed small V_b_. The red and green arrows represent the thermionic current and tunneling current, respectively. The solid and dashed lines indicate the major contribution and minor contribution, respectively. Reproduced with permission.^[^ ^47^ ^]^ Copyright 2022, American Chemical Society.

Tunneling transistors mainly rely on quantum tunneling, which means electrons can directly travel through potential barriers in a certain condition.^[^ ^45^ ^]^ This phenomenon is obvious in graphene because its special band structure at the Dirac point allows electrons to easily tunnel, which will hamper the high on‐off ratio of the device. Therefore, Britnell et al. first reported a field‐effect tunneling transistor based on graphene‐hBN‐graphene heterostructures,^[^ ^46^ ^]^ where hBN acts as an insulating layer to suppress the tunneling current at off state and achieve a higher on‐off ratio. The schematic can be seen in **Figure** **2**a. Three dominant factors for the enlarged on‐off ratio can be concluded as: i) V_g_ can lift Fermi level in graphene, increased Fermi level in both graphene electrodes then lead to a reduction in Δ (effective barrier height, determined by the band offset between the graphene and hBN as a function of V_g_.) (Figure 2b). ii) Bias voltage (V_b_) can penetrate through graphene and alter the shape (decrease the width) of the insulating barrier (Figure 2d). iii) tunneling density of states (DoS) will increase as the Fermi level moves away from the neutral point in graphene. The relationship between tunneling conductivity and DoS can be given by:

(1) $$\sigma_{T}\propto \mathrm{Do}S_{B}\left(V_{g}\right)\times \mathrm{Do}S_{T}\left(V_{g}\right)\times T\left(V_{g}\right)$$

Where T(V_g_) is the transmission coefficient through the hBN barrier. This very first vertical graphene tunneling transistor has certain advantages and drawbacks: i) In terms of advantages, the transit time for tunneling is extremely short, making the field effect tunneling transistor ideal for high‐speed operations; ii) In terms of disadvantages, this device is hindered by its direct tunneling mechanism, and the limited changes in the Fermi level of graphene modulated by V_g_ (typically, <0.5 eV, limited by dielectric breakdown) are still small compared with the large barrier height (≈1.4 eV), resulting in a significantly low on‐state current density (typically in the order of 10–100 pA µm^−2^), consequently, rendering it impractical for real‐world applications. With the development of material science, the junction‐based transistor utilizing graphene and 2D materials, especially TMDs has been extensively studied. In order to further improve on–off ratio and on‐state current, recently, Bai et al. made use of 10 nanometers (15 layers) WS_2_ (Tungsten disulfide) as an insulating layer and adopted van der Waals (vdW) heterostructures to fabricate vertical vdW graphene–WS_2_–graphene (GWG) tunneling transistor, which will be introduced in following part.

The schematic of this GWG transistor can be seen in Figure 2f.^[^ ^47^ ^]^ This transistor shows a relatively small off current and a large on‐off ratio of 1.5 × 10^6^ at room temperature (Figure 2g), which performs 4 orders of magnitude higher than Britnell et al. work.^[^ ^46^ ^]^ The enhanced on‐state current owes to three aspects: i) WS_2_ has the highest carrier mobility at room temperature among all XS_2_. ii) WS_2_ can naturally decrease barrier height due to a small band offset between WS_2_ and graphene (≈0.4 eV) compared with a high barrier height between hBN (≈1.4 eV). By applying V_g_, barrier height can be further modulated down to ≈0.1 eV. Since barrier height is quite low, thermionic emission enhanced larger on‐state current can be found even at room temperature, and the difference can be more obvious when a V_g_ is applied to further modulate barrier height, which helps improve on‐state current limited by only tunneling in typical tunneling FETs. iii) vdW heterojunction can achieve an atomically clean and electronically sharp interface, which will reduce the Fermi level pinning effect and reduce the tunneling current path leaking.^[^ ^48^ ^]^ By taking the temperature dependence experiment, Bai et al. successfully found different degrees of temperature dependence for conductance under different temperature regions, which is again a demonstration of the existence of thermionic emission. In addition, Bai et al. discovered different dominant transport mechanisms (thermionic emission or tunneling) under different V_g_ (Figure 2i).

### 2.2 Tunable Schottky Barrier Transistor

**Figure 3:**
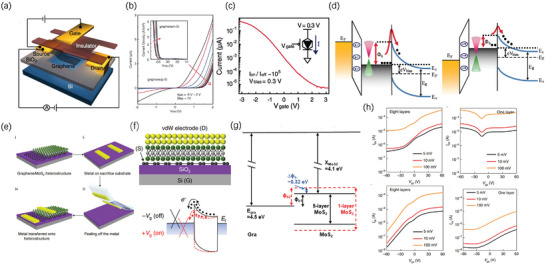
a) Schematic of the first tunable Schottky barrier transistor. b) The current and bias voltage characteristics of p‐type and n‐type device characteristics. c) The forward diode current as a function of gate V_g_ at fixed V_b_ = 0.3 V. d) Schematic energy band diagrams of graphene‐silicon junction under negative and positive V_b_. Reproduced with permission.^[^ ^50^ ^]^ Copyright 2012, American Association for the Advancement of Science. e) Corresponding fabricating process of sub‐1 nm vdW vertical transistor. f) Cross–section schematic of the device and band diagram for thermionic emission in on and off state. g) In the enlarged bandgap of MoS_2,_ red dash represents monolayer MoS_2_ and the black line represents multilayer MoS_2_. h) *I*_ds_–V_gs_ transfer curves of evaporated electrode (top) and vdW electrode transistor (bottom) for eight‐layer channel length and one‐layer channel length, respectively. Reproduced with permission.^[^ ^48^ ^]^ Copyright 2021, The Author(s), under exclusive license to Springer Nature Limited.

Solid silicon electronics have a certain drawback that hinders high performance in silicon electronics^[^ ^49^ ^]^: a Schottky barrier would exist between the silicon and metal and remain constant due to the Fermi‐level pinning effect caused by surface states introduced during the fabrication process. Therefore, the first tunable Schottky barrier transistor based on a graphene‐silicon junction was invented by Yang et al.^[^ ^50^ ^]^ (See schematic in **Figure** **3**a) and a relatively large on–off ratio of 10^5^ was achieved (Figure 3b) in this device. The core mechanism of the tunable Schottky barrier lies in two aspects: i) surface states can be suppressed in graphene‐silicon heterojunction due to the inert chemical property of graphene thus alleviating the Fermi pinning effect. ii) graphene's work function can be tuned over a wide range via the electrostatic field effect. In Yang et al. experiment, the key lies not only in taking advantage of graphene's intrinsic chemical property but also in a successfully achieved atomically sharp interface between graphene and hydrogenated silicon by CVD (chemical vapor deposition). To achieve current flow in this structure, applying negative V_g_ can induce holes in graphene, increasing its work function and increasing the Schottky barrier height, therefore, the current across the Schottky barrier decreases, while positive V_g_ decreases the Schottky barrier height and increases reversed current (Figure 3d).

Although Yang et al. have already achieved a relatively clean interface between graphene and silicon. However, in order to attain an ultrashort vertical transistor with a tunable Schottky barrier, metal atom diffusion, defects and disordered interfaces caused by metal deposition in source/graphene junction are still unacceptable.^[^ ^51^, ^52^, ^53^ ^]^ These factors reduce the effective thickness of the vertical channel and increase the tunneling current.^[^ ^54^, ^55^ ^]^ Besides, additional surface states can be inevitably introduced to the vertical channel after metal electrode deposition, which will weaken the gate's modulation.

Recently, by making use of the vdW integration, Liu et al. demonstrated the sub‐1 nm vertical channel FETs.^[^ ^48^ ^]^ MoS_2_ flake is first mechanically exfoliated and dry transferred on top of graphene, the Ag electrodes are then mechanically laminated and transferred on top of MoS_2_/graphene vertical heterostructure (Figure 3e). This kind of transistor's operation mainly relies on thermionic emission, the barrier height formed between graphene and MoS_2_ can be modulated by V_g_ from 3.6 to 96 meV (3.6 nm thick device), which is quite small. A linear output relationship of *I–V* curves (no obvious rectifying effect) can be observed at on state (Figure 3f). While tunneling plays an important role in device operation when the channel is extremely short because in this case, the enlarged MoS_2_ bandgap will increase the Schottky barrier, thus greatly suppressing thermionic emission (Figure 3g). Liu et al. also demonstrated a larger on–off ratio of ≈10^3^ with a channel length of 5 nanometers (8 layers) compared to evaporated metal VFETs (Figure 3h). The monolayer MoS_2_ channel (0.65 nm) transistor, still shows an n‐type behavior and a current on–off ratio ≈ 26, while the evaporated top electrode transistor shows a bipolar behavior (just like a graphene channel transistor) and a current on–off ratio <5, which indicates that the monolayer MoS_2_ is severely influenced by top diffusive metal atoms, so the MoS_2_/graphene junction is totally dominated by the bottom graphene electrode contact, which again indicates the superior of vdW integration especially in fabricating ultrasmall vertical transistors.

### 2.3 Hot Electron Transistor

**Figure 4:**
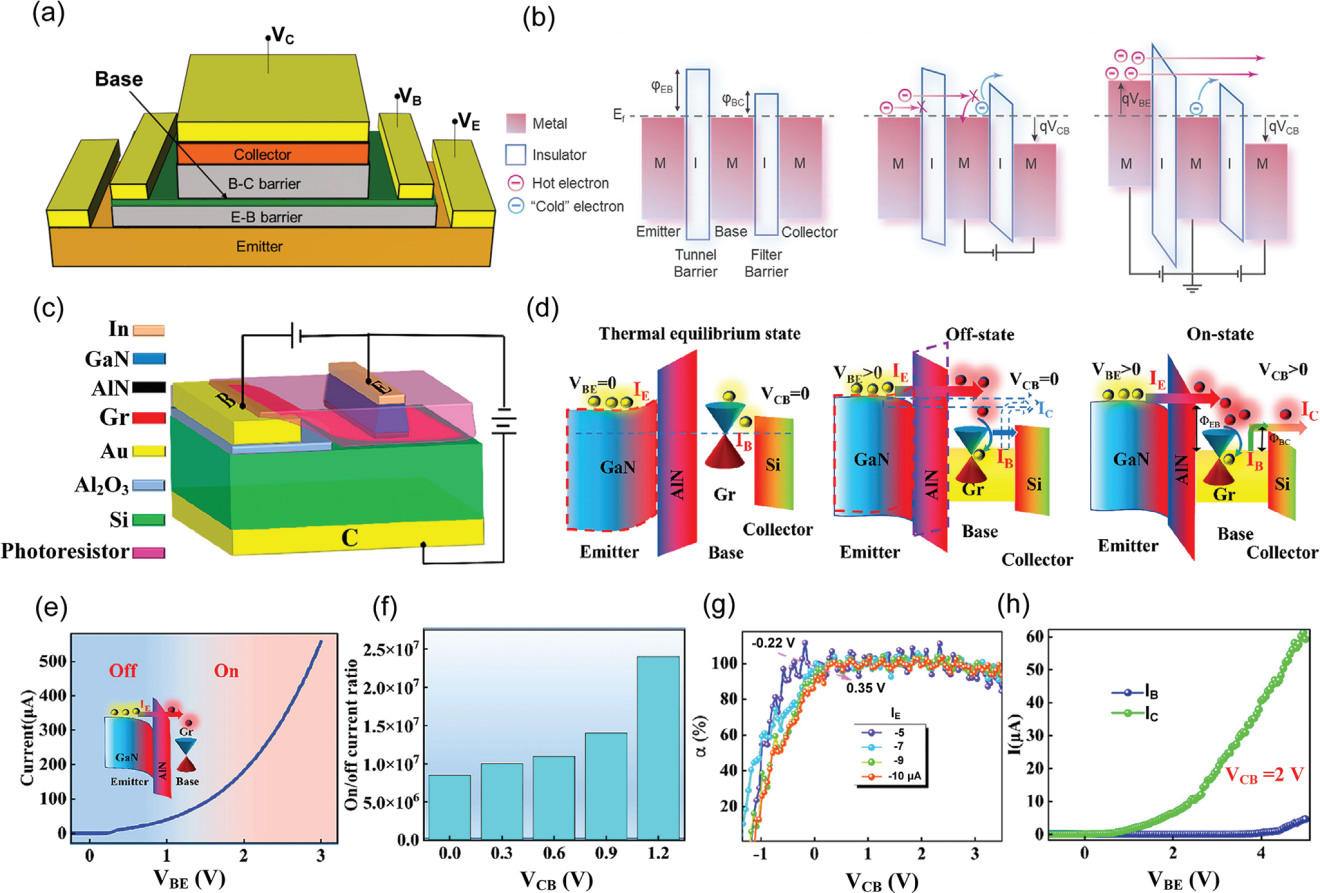
a) Basic structure of a hot electron transistor. Reproduced under the terms of the CC‐BY license.^[^ ^37^ ^]^ Copyright 2018, by the authors. b) Schematic of energy band diagram for hot electron transistor in different states. Reproduced under the terms of the CC‐BY license.^[^ ^67^ ^]^ Copyright 2021, Author(s). c) Basic structure for newly designed hot electron transistor_._ d) Schematic view of energy band diagram under different states for this new hot electron transistor. e) High current emission density in B–E junction at on state. f) Current on–off ratio ≈10^7^. g) Biasing the device in the common‐base mode thus gains a high value of *α*. h) Biasing device in the common‐emitter configuration thus gains a value of *β*. Reproduced with permission.^[^ ^68^ ^]^ Copyright 2023, American Chemical Society.

Hot electron transistor (HET) is a three‐terminal heterostructure device where the ultra‐thin base layer is sandwiched between two thin insulating barriers (base‐collector barrier and base‐emitter barrier). Hot electron transistor was first put forward by Mead^[^ ^59^ ^]^ in order to overcome minority carrier storage time in bipolar transistors. This kind of transistor has developed a lot, but the basic principle remains similar although layer structure and the material might change. The first hot electron transistor is structured as metal‐insulator‐metal‐insulator‐metal, here we will put a basic device structure (**Figure** **4**a), band diagram (Figure 4b) and illustrate the operation principle of a metal‐insulator‐metal‐insulator‐metal structure transistor, and also suitable for other hot electron transistors.

When V_CB_ > 0 and V_BE_ = 0, few electrons can flow from the emitter to the base because of the large insulator barrier at the emitter‐insulator‐base junction. Also, few electrons can flow from collector to base due to a properly designed collector‐base barrier. The device is in an off state (To mention that, a small number of cold electrons in the base can still travel to the collector either by thermionic emission or tunneling, which will cause leakage current).

When *V*_CB_>0 and *V*_BE_>0, electrons can tunnel from the emitter, travel through the insulating layer, and gain energy to become hot electrons (high‐energy electrons) in the base. It's important that the injected electrons from the emitter to the base need to have much higher energy compared to the electrons’ thermal population (cold electrons) in the base (For example, if the base is a metal, then the hot electrons need to gain energy >*E*_C_,^[^ ^60^ ^]^ if the base is a semiconductor, then the hot electrons need to have energy >*E*_F_.^[^ ^37^ ^]^ Under this scenario, when hot electrons and cold electrons are approaching the same potential barrier, hot electrons are more likely to cross the barrier. In addition, due to the high velocity of injected electrons, base transit time is only a few tenths of a picosecond, which is the key for high‐frequency HETs, and this will bring up a topic that minimizing the thickness of the base can help electrons traveling through base ballistically, which is a critical way to improve performance of HET. Once electrons have traveled through the base, they will reach the base‐collector barrier (filter barrier). The base‐collector barrier is designed to prevent leakage current caused by cold electrons through either thermionic emission or tunneling as much as possible and allowing most of the hot electrons to pass through the potential barrier. However, if the injected electrons from the emitter to the base do not have enough energy, they will bounce back to the base, become cold electrons again, and cause base current *I*_B_.

Common‐base current transfer ratio *α* = *I*_C_–*I*_C_(leakage)/*I*_E_ and the common‐emitter current gain *β* = *I*_C_/*I*_B_ are important merits for the DC operation of HET. At an idealized condition, *α* ≈ 1 and *β* as large as possible are needed. For the very first hot electron transistor, *α* is extremely low (0.1) due to the bad quality of base metal and barrier oxides though the barrier (Al_2_O_3_) was already thin enough for tunneling. Since the first hot electron transistor was invented, many optimizations were implemented to achieve better performance^[^ ^61^, ^62^, ^63^ ^]^ by trying different materials and layer structures, and the main mechanisms for optimizing are shown in the following part.

From the perspective of emitter‐base barrier, since tunneling probability is inversely and exponentially proportional to the barrier height and thickness^[^ ^64^ ^]^ and a large *V*_BE_ widens the electron distribution function due to optical and acoustic phonon scattering in the insulator and degrades *α*,^[^ ^42^ ^]^ so it is important to use a barrier material which is properly thin and has proper potential difference (barrier height) with emitter material to gain enough high *α* and can be easily turned off. In addition, the E–B barrier must be higher than and similar to the B–C barrier (*φ*_EB_ ⪆ *φ*_BC_),^[^ ^65^ ^]^ otherwise, the *α* will be extremely low^[^ ^66^ ^]^ because many hot electrons will be reflected by the filter barrier (B–C barrier).

From the perspective of base material, since a high *α* is wanted, so base needs to be ultimately thin to achieve ballistic transport of electrons (base length should be shorter than electron mean free path) to minimize scattering effects while maintaining low intrinsic–extrinsic base resistance. However, for most bulk materials, reducing their thickness would cause serious structural defects. Since 2D materials own great conductance and can be scaled down to one layer thickness, they have become candidates for further optimizing HET.

From the perspective of the base–collector barrier,^[^ ^67^ ^]^ this barrier is critical and should be carefully controlled, which means that the B–C barrier must be low/thin enough (especially, lower than the E–B barrier) to avoid reflecting lots of hot electrons while high/thick enough to prevent leakage current caused by cold electrons.^[^ ^42^ ^]^

Here, we will introduce a recently designed hot electron transistor^[^ ^68^ ^]^ based on GaN‐AlN (<10 nm)‐one layer graphene ‐Si junction, which can achieve a record DC gain (*β*) of 16.2, *α* close to the limit of 99.9%, a large emitter current density of ≈68.7 A cm^−2^, a high on–off current ratio reaching ≈10^7^ which is higher than theoretical predicts^[^ ^69^ ^]^ of a graphene‐based hot electron transistor.

In this device, Zou et al. designed a transistor with a GaN‐AlN high‐barrier injection layer as the emitter, single‐layer graphene as the base, and a low‐barrier shield layer of an n‐doped Si as the collector (Figure 4c). By only considering B–E junction with two applied terminal voltages, an emission current density can be found ≈68.7 A cm^−2^ at *V*_BE_ = 3 V (Figure 4e), this high injection current owes to a small conduction band offset at the junction, small lattice mismatch and high‐quality trap‐free interface between GaN/AlN. The transport mechanism in the B–E junction can be demonstrated for either direct tunneling or Fowler‐Nordheim tunneling under different values of *V*_BE_ (Direct tunneling dominates when *V*_BE_ is smaller than 1.13 V, Fowler‐Nordheim tunneling dominates when *V*_BE_ is higher than 1.13 V). The B–C junction shows both tunneling and thermionic emission for hot electrons, which relies on the controllable Schottky barrier formed between graphene and silicon. It's noted that this B–C junction allows a lower tunneling voltage due to a smaller band offset between graphene and silicon, this configuration can allow the device to work at a lower voltage and suppress thermal electron scattering. Then, Zou and his co‐workers biased the device in the common‐base mode, since *I*_C_ is determined by the modulation of the filter barrier of *V*_CB_, so changing *V*_CB_ can gain a different *I*_C_ at a given *I*_E_ and finally get a max *α* of 99.9% (Figure 4g). Then they biased the device in the common‐emitter configuration and extracted a fixed *V*_CB_ so that the leakage current and* I*_B_ are fixed. Since *I*_C_ is also determined by *V*_BE_, so changing *V*_BE_ can obtain different *I*_C_ and finally get a max *β* ≈16.2 (61.8/3.7 µA) (Figure 4h). To conclude, Zou et al improved hot electron transistor by optimizing the junction area (making the collector more conducive for electrons), achieving a high‐quality junction interface, and using a steep Schottky barrier formed between base‐collector, the parameters are better than the prototype graphene hot electron transistor designed by Zeng et al.^[^ ^42^ ^]^ in 2013 and recently designed GHET by Xu et al.^[^ ^70^ ^]^ in 2019 while exceeding theoretical performance predicts.

## 3 Nano‐gate Transistor

Si‐based transistors are approaching their scaling limits, from 2D devices to FinFET to gates all around the structure, few of them can further scale gate length below 5 nm. Besides, SCEs are also severely impacting the scaling pace while continue using Si as the material. It is of great importance to find other materials and design new structures to ultimately scale the device. The following part will first introduce SWCNT‐gated transistors, making use of the SWCNT's diameter as effective gate length and reaching sub‐5 nm gate length limit. Then a sub‐1 nm gate length graphene‐gate transistor will be introduced and the final part will be nanowire gate transistors. These devices have relatively good resistance to SCEs while achieving small gate lengths at the same time.

### 3.1 Sub‐5 nm CNT Nano‐gate transistors

**Figure 5:**
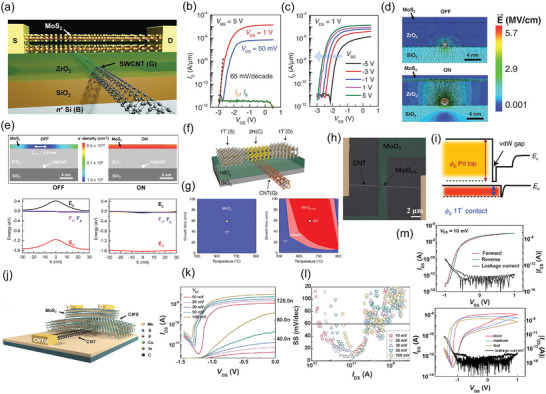
a) Schematic view of 1 nm SWCNT gate transistor. b) The *I*_DS_—V_gS_ characteristics. c) The *I*_DS_–*V*_BS_ characteristics at *V*_DS_ = 1 V illustrate the effect of Si back‐gate bias on the extension region‘s resistance. d) The simulated electric field at on–off states in the device. e) Simulation figure of electron density at on and off state with corresponding energy band diagrams. Reproduced with permission.^[^ ^71^ ^]^ Copyright 2016, American Association for the Advancement of Science. f) Chemically synthesized 1T′/2H MoTe_2_ FET with a CNT gate. g) Tellurization conditions for phase evolution. h) SEM image of the channel and source‐drain electrodes deposited with MoO_2.0‐2.5_ and MoO_3_ patterns, respectively. i) Schematic view of barrier heights (*Φ*_b_) for FETs with a Pd contact and a 1T′ contact. Reproduced with permission.^[^ ^75^ ^]^ Copyright 2019, The Author(s), under exclusive license to Springer Nature Limited. j) Schematic view of 4.3 nm gate length transistor with FE dielectric. k) Transfer characteristics for 4.3 nm gate length transistor. l) SS of 4.3 nm gate length transistor for both forward sweep and reverse sweep direction. m) Top‐gated long channel device fabricated in this work shows smaller anticlockwise hysteresis (top) compared to the 4.3 nm transistor (bottom). Leakage current is shown as black lines. Reproduced with permission.^[^ ^77^ ^]^ Copyright 2020, Science China Press. Published by Elsevier B.V. and Science China Press. All rights reserved.

In 2016, Desai et al. first achieved a 1 nm gate length scaling limit by making use of ultrasmall diameter of carbon nanotube (CNT) as a local gate electrode.^[^ ^71^ ^]^ The device structure is shown in **Figure** **5**a. To fabricate this transistor, CNT grown by CVD will be first transferred and located onto a Si/SiO_2_ substrate, followed by contacting with palladium via lithography and metallization. Then, ZrO_2_ dielectric will be deposited to cover CNT and MoS_2_ will be transferred to ZrO_2_, finally Nickel source and drain contacts will be made to MoS_2_ channel. MoS_2_ is adopted as the channel material due to its larger effective electron mass^[^ ^72^ ^]^ and lower dielectric constant^[^ ^73^, ^74^ ^]^ compared with Si, which is beneficial for suppressing SCEs. In this device, the silicon back gate (*V*_BS_) is significant, since it is used to strongly invert the MoS_2_ extension region even to *n* ^+^ state and reduce the region's resistance. Transfer curve with fixed *V*_BS_ of a bilayer MoS_2_ device shows a near ideal subthreshold swing (SS) of ≈65 mV dec^−1^ at room temperature, a current on–off ratio of ≈10^6^, and the DIBL of ≈290 mV V^−1^ (Figure 5b), while the transfer curve with varying *V*_BS_ (Figure 5c) shows clearly that, an increased *V*_BS_ leads to heavier inversion, resulting in higher on‐state current, smaller SS, resistance, and threshold voltage. Detailed TCAD simulation is done to explore the mechanism and determine the effective channel length of such a buried CNT gate transistor. The electric field contour in device at on and off states is shown in Figure 5d. When the CNT gate voltage is negative, electrons above the gate will be driven away by electric field force, *E*_C_ above the CNT gate will bend upward, away from the Fermi level, thus forming a low‐density (smaller than threshold density) electron channel area with an effective channel length of 3.9 nm (Figure 5e). When the device is in on state, since the device has already been inverted by *V*_BS_, combining the downward bending trend of *E*_C_, *E*_C_ across the whole channel will be close to Fermi level (Figure 5e), so the whole channel has a high electron density, the effective channel length can be simply regarded as gate length, namely ≈1 nm.

In 2019, Zhang et al. further adopted heterophase engineering to fabricate a transistor with ≈4 nm length CNT gate.^[^ ^75^ ^]^ The device structure can be seen in Figure 5f. In the experiment, precursors (MoO_x_) will be first lithography‐patterned into desired structures according to device design, then, by controlling the tellurization condition (Figure 5g), MoO_3_ will be changed into metallic 1T′ phase MoTe_2_ as drain/source electrode, while MoO_2.0‐2.5_ will be changed into semiconducting 2H phase MoTe_2_ as channel. This 4 nm gate device fabrication flow can be briefly concluded as follows: CNTs arrays are buried under the ALD‐grown HfO_2_ dielectrics, then the channel and source‐drain electrodes are deposited with MoO_2.0‐2.5_ and MoO_3_ patterns, respectively (Figure 5h). Finally, the samples are tellurized to convert the precursor oxides to semiconducting 2H MoTe_2_ and metallic 1T′ MoTe_2_, respectively. The obtained FETs with ≈4 nm CNT gates exhibit excellent switching characteristics with an SS of ≈73 mV dec^−1^ and an on–off current ratio of ≈10^5^. Note that, this one‐step synthesizing method replaces the physical channel‐electrodes contacts with covalent connections, dramatically improving charge injection and reducing contact resistance (measured barrier height is ≈ 20 meV for 1T′ contact compared with ≈200 meV for Pd contact, Figure 5i). What's more, transistors fabricated by this heterophase engineering method are not the first time. In 2016, a 7.5 nm channel length back‐gate transistor was fabricated by using different phases of MoS_2_ as channel/electrodes.^[^ ^76^ ^]^ The channel length and electrode length in this work are both determined by the period *L*_o_ of block copolymer PS‐b‐PDMS. In simple terms, PS‐b‐PDMS works as a mask on MoS_2_, after selective plasma etching for PS, the exposed MoS_2_ under PS will be changed into a metallic phase as electrodes, while the unexposed MoS_2_ under PDMS will remain at a semiconducting phase as channel, the length of PDMS (channel) and electrode length are both equal to half *L*_o_, 7.5 nm. The resistance at the 2H‐1T′ interface is found to be 75 Ω µm, again indicating a dramatically decreased resistance in the heterophase junction. Though this paper did not adopt CNT as a gate, it provided a possible way for simultaneously achieving small channel length and electrode length, combining Zhang et al.‘s work, a transistor with ultimately scaled gate/channel/electrode length while maintaining a low contact resistance is quite possible.

In 2020, Wang et al. first time explored 2D FET combined with both a subthermionic switch and a sub‐5 nm gate length.^[^ ^77^ ^]^ In this work, a ≈4.3 nm (diameter) CNT is adopted as a gate, CuInP_2_S_6_ (CIPS), a recently found ferroelectric (FE) material, is chosen as gate dielectric due to its good ferroelectricity at a few nanometers thickness, making it appropriate for ultrashort gate length transistor. Moreover, CIPS can function well as a gate voltage amplifier due to its negative capacitance,^[^ ^78^, ^79^ ^]^ thus achieving SS smaller than 60 mV dec^−1^ at room temperature. To fabricate this device, two electrodes are deposited on CVD‐grown CNT as gate terminal, then CIPS and MoS_2_ channels are exfoliated and stacked to form vdW heterojunctions. Finally, the source and drain terminal are made, the schematic view can be seen in Figure 5j. Since negative capacitance FETs relying on ferroelectric modulation always suffer from charge storage‐induced hysteresis, adopting vdWHs is beneficial for excellent contact between MoS_2_ and CIPS, including fewer interfacial trap states, a stronger combination between FE insulator and channel, which theoretically enables an anti‐hysteresis property. The transfer curve indicates that ≈4.3 nm gate can still turn on–off the device with a ratio of ≈10^5^ (Figure 5k) together with an obvious subthermionic switching behavior independent of V_DS_ (Figure 5l, minimum SS reaches down to 6.1 mV dec^−1^). However, a larger anticlockwise hysteresis can be observed compared to the planar, top‐gated long channel device fabricated during experiment (Figure 5m). Notably, smaller SS and larger anticlockwise hysteresis in a 4.3 nm transistor are contradictory to the result from former numeric simulation^[^ ^80^, ^81^ ^]^ probably attributed to dramatically decreased capacitance of CIPS When the gate length is greatly scaled down to 4.3 nm, the device will more easily operate in a region where the absolute value of FE capacitance is smaller than that of channel. Therefore, more works are needed to figure out the capacitance matching issue in the small feature‐size situation.

### 3.2 Sub‐1 nm Graphene Nano‐Gate Transistor

Although Desai et al. have reached a 1 nm gate length limitation, it is hard to further scale gate length below 1 nm by using a SWCNT gate due to the limitation of its diameter. As the thinnest conductive material, graphene has gained wide attention and has been used as drain/source electrodes^[^ ^82^, ^83^, ^84^, ^85^ ^]^ or channel material^[^ ^86^, ^87^ ^]^ for many transistors. However, using graphene as the gate electrode for a transistor while achieving a nano‐scale device is very rare. Many graphene‐gated transistors adopt a planar structure, for instance, graphene can be adopted as a gate to form MESFET^[^ ^88^, ^89^ ^]^ and devices are limited at the micron‐scale, which means that the natural thickness of graphene has not been fully explored in scaling transistor size. Nevertheless, in 2020, Ren et al. successfully found a way to approach the ultimate scaling limits by adopting a buried graphene gate in a sidewall‐structured transistor with a physical gate length of 0.34 nm, an effective channel length of 0.34 nm at on‐state and 4.54 nm at off‐state.^[^ ^6^ ^]^ This transistor not only achieves scaling limits but also has a desirable on–off ratio which is larger than 1.02 × 10^5^ and an SS equals 117 mV dec^−1^.

**Figure 6:**
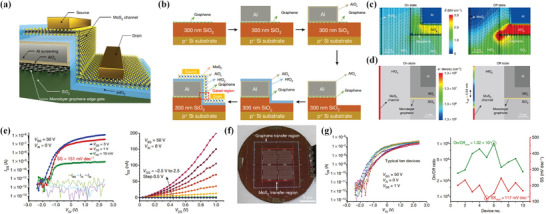
a) Schematic view of sub‐1 nm gate length transistor. b) Fabrication flow of sub‐1 nm transistor. c) Simulation results of electric field contour in on and off state. d) Simulation result of electron density in channel in on and off state. e) Transfer (left) and output (right) characteristics of a typical device. f) Optical image of a 2‐inch wafer‐scale fabrication of the sub‐1 nm side‐wall transistors. g) As fabricated 49 transistors show a small variation of device parameters. Reproduced with permission.^[^ ^6^ ^]^ Copyright 2022, The Author(s), under exclusive license to Springer Nature Limited.

The device's Schematic view and the corresponding fabrication flown can be seen in **Figure** **6**a,b, respectively. Graphene is adopted as the gate due to its high electrical conductivity^[^ ^90^, ^91^ ^]^ and ultrathin body; aluminum is used to screen the vertical electric field from the upper surface of graphene. Due to the screening effect of aluminum and confinement of side wall structure, only the electric field from the edge of graphene can effectively modulate the MoS_2_ channel; while graphene is one atom layer thick (0.34 nm), so the gate length can be greatly scaled. A TCAD simulation is further conducted to determine the effective channel length. By observing that the effective gated electric field comes from the edge of graphene at off state (Figure 6c, Si back gate *V*_BS_ is fixed at 50 V, Al is fixed at 0 V), a 0.34 nm gate length can be demonstrated. By defining the effective channel length as the channel region with electron density *N* < *N*_threshold_ = 1.3 × 10^5^ cm^−2^ (Figure 6d): i) at off‐state, a 4.54 nm side‐wall MoS_2_ channel which is close to graphene's edge can be determined as the effective channel length; ii) at on‐state, since the whole channel has a high electron density, gate length can be directly regarded as the effective channel length, which equals to 0.34 nm. According to the electrical characterization of a typical device, the SS value is 151 mV dec^−1^ at V_DS_ = 1 V, on–off ratio reaches up to 3.44 × 10^5^ at V_DS_ = 3 V and the DIBL effect is ≈126 mV V^−1^ (Figure 6e). In addition, this side‐wall transistor can be fabricated on a wafer scale (Figure 6f) and as fabricated 49 devices show uniformity in transfer curves, on–off ratio, and SS (Figure 6g).

### 3.3 Nanowire Gated Transistor

In addition to CNT and graphene, nanowire is being actively pursued as a channel material for GAA (gate‐all‐around) transistors,^[^ ^92^, ^93^, ^94^ ^]^ which are intended to enhance device performance. But in this part, some nanowire‐gated transistors will be introduced, all of them use the diameter of the nanowire as channel length and have desirable electric properties.

#### 3.3.1 Multiple NW Gates Transistor

**Figure 7:**
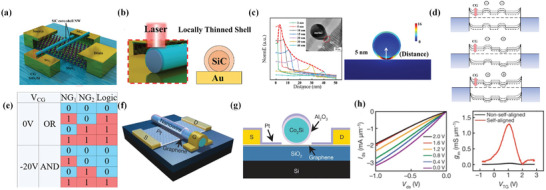
a) Schematic of locally thinned, core−shell nanowire double gates transistor. b) Schematic of interface between nanowire and external Au electrode after laser irradiation. c) Normalized energy field distributions along nanowire diameters with different SiO_2_ shell thicknesses under the same laser irradiation (left), simulation of the energy‐field distribution when the thickness of the shell is 5 nm (right). d) Energy band diagram when *V*_CG_ and two NGs are all applied. e) Logic of the device in different situations. Reproduced with permission.^[^ ^95^ ^]^ Copyright 2023, American Chemical Society. f) Schematic view of high‐speed graphene transistor with a self‐aligned nanowire gate. g) Deposited Pt thin film is separated by nanowire. h) Enhanced performance of self‐aligned structure. Reproduced with permission.^[^ ^97^ ^]^ Copyright 2010, Springer Nature Limited.

In 2022, Xiao et al. found a way to integrate double nanowire gates into one MoS_2_ channel transistor.^[^ ^95^ ^]^ The schematic view of this double‐gate transistor can be seen in **Figure** **7**a. In this transistor, n‐type nanoflake MoS_2_ serves as a channel, and the Si in Si/SiO_2_ substrate serves as a global control gate (CG). Additionally, to apply nanowire gate voltage individually, each core‐shelled SiC nanowire gate (NG) needs an external electrode, in which the SiO_2_ will be locally thinned/removed by femtosecond laser irradiation in the contact region between semiconducting SiC and external electrode (Figure 7b), while the remaining part SiO_2_ shell will keep intact, to protect against short circuiting between NGs. In the experiment, Xiao et al. found that a 5 nm core–shell can maximally enhance laser power in the contact part between the nanowire and electrode^[^ ^96^ ^]^ (Figure 7c) and will cause less damage to the core.

Since this MoS_2_ transistor integrates double nanowire gates with a control back gate, the effect of different gates can be important. When voltages are applied to two NGs simultaneously, the schematic of energy band can be seen in Figure 7d. It was found that the potential area around the nanowire gate will be strictly confined and only the energy band below it will be modulated, having little influence on the relatively far area, which means that the influence region of two NGs won't intersect and NGs can individually control the energy band. What's more, the Schottky barrier between channel and electrode is found to be out of reach for NGs; therefore, CG can be additionally applied to modulate the barrier height, thus influencing the overall device current. By making use of this special merit, this single transistor can achieve both AND and OR logic. i) When CG is set to logic 0 (negative voltage), both NGs need to be set to logic 1 to lift the overall device current, which corresponds to logic AND; ii) When CG is set to logic 1 (0 V), the device can be turned on with only one logic 1 input, which corresponds to logic OR (Figure 7e). It's also worth noting that, this device has a relatively large SS (for both gates, >450 mV dec^−1^) and low on state current, which mainly attributes to a high Schottky barrier height (≈0.9 eV) and damage caused by laser irradiation. Nevertheless. this multifunctional deice provides the potential for low dimensional material‐based transistors in further reducing transistor numbers in building logic circuits, thus greatly reducing the overall footprint area.

#### 3.3.2 Self‐aligned NW Gate Transistor

Self‐alignment technology is an effective way to eliminate damages caused by lithography and scale the effective channel length, thus greatly reducing parasitic capacitance and resistance. Since most CNT‐gated transistors and NW transistors mentioned above suffer from large physical distances between electrodes compared to gate length, achieving a self‐aligned structure is an alternative way to improve the device's performance. In this section, a groundbreaking work by Liao et al. will be introduced, in which a NW gated transistor with a self‐aligned structure is achieved for the first time.^[^ ^97^ ^]^

In this work, self‐aligned transistors with channel lengths ranging from 100 to 300 nm are fabricated. The schematic view of a typical device can be seen in Figure 7f. To fabricate the device, graphene is first peeled onto a Si/SiO_2_ substrate, then Co_2_Si─Al_2_O_3_ core‐shell nanowire is physically dry transferred onto graphene, followed by electron‐beam lithography, shell etching to expose the Co_2_Si core (to connect the external electrode), and the metallization process to define the external source, drain, and gate electrodes. Finally, thin‐layer platinum will be deposited on top of the whole graphene and across the nanowire; thus, platinum will be separated by the nanowire gate (Figure 7g), forming a self‐aligned structure. Electrical testing shows that a self‐aligned configuration can greatly enhance a transistor's performance, including a higher on‐state current (3.32 mA µm^−1^ for a 140 nm channel length transistor) and a higher transconductance (1.27 mS µm^−1^) (Figure 7h).

Additionally, Liao et al. explored the intrinsic cut‐off frequency of this device. The 140 nm transistor shows an intrinsic cut‐off frequency of 300 GHz, which is mainly attributed to the small gate capacitance and increased transconductance due to the small gate length and self‐aligned configuration, respectively. This high intrinsic cut‐off frequency also benefits from the dry transfer method, which causes few defects in the graphene lattice compared to the common dielectric deposition method, thus leading to less degradation of graphene's electron mobility. However, this kind of high‐speed transistor can still be limited or suffer from: i) high gate delay compared to such high speed. ii) low extrinsic cut‐off frequency (2.4 GHz for a 144 nm gate length transistor) due to the large ratio between the parasitic and gate capacitances. It's worth noting that, after this work's publication, many self‐aligned transistors with nanoscale channel lengths and optimized performances have been proposed and will be introduced in the following section.

## 4 Nanofabrication for Small Feature‐size Transistor

With the rise of new materials and new device structures, the development of nano‐scale fabrication can also be important. This part will mainly focus on the emerging advanced fabrication techniques for nano‐scale transistors.

### 4.1 Nano‐Scale Self‐Aligned Transistor

#### 4.1.1 Resolution‐independent Self‐Aligned Structure

For a normal self‐aligned method, the gate will first be deposited on gate oxide, then the oxide will be etched to form two windows for later impurity implantation, which will act as source and drain regions. Under this scenario, the gate will be automatically aligned with the source and drain electrodes, and the performance of the whole device can be improved. However, this method mostly limits the device's channel length at the micron scale.^[^ ^98^, ^99^ ^]^ With the development of 2D materials such as graphene and MoS_2_, the self‐aligned method can also be improved to achieve an optimized self‐aligned structure.

**Figure 8:**
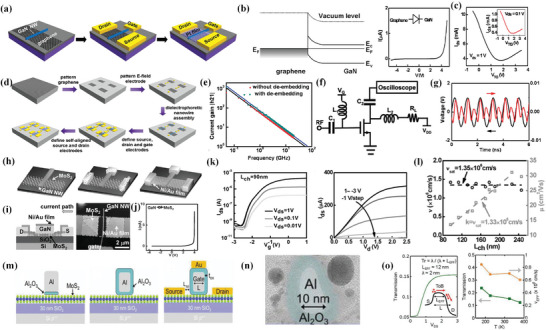
a) Fabrication flow of self‐aligned GaN nanowire gate transistor. b) Schottky barrier formed between graphene channel and GaN gate (left), rectification effect of this Schottky barrier, which is helpful in preventing leakage current form channel to the gate. c) Transfer characteristics at *V*_DS_ = 1 V for the GaN gate transistor. The inset shows the transfer characteristics at *V*_DS_ = 0.1 V. Reproduced with permission.^[^ ^100^ ^]^ Copyright 2010, American Chemical Society. d) Fabrication flow of large‐scale self‐aligned transistors on glass substrate. e) The small‐signal current gain |h_21_| as a function of frequency *f* at *V*_DS_ = 1 V, highlighting an intrinsic cut off frequency of 72 GHz, and an extrinsic cut off frequency exceeding 50 GHz without the de‐embedding process (including the exact pad layout as “open” and “short” test structures). f) Circuit diagram of a radio frequency doubler based on single self‐aligned transistor. g) Measured input (black) and output (red) signals of the frequency‐doubling circuit when the graphene device is gated near the Dirac point. Reproduced with permission.^[^ ^101^ ^]^ Copyright 2011, American Chemical Society. h) Fabrication flow of self‐aligned MoS_2_ channel transistor. i) Structure of self‐aligned MoS_2_ transistor. j) Schottky barrier formed between MoS_2_ and GaN, with a ≈3 V turn‐on voltage. k) Transfer characteristic curve (left) and output characteristic curve (right). l) The changes of the V_drift_ with varying channel length. Reproduced with permission.^[^ ^102^ ^]^ Copyright 2016, WILEY‐VCH Verlag GmbH & Co. KGaA, Weinheim. m) Fabrication flow of 10 nm self‐aligned transistor. n) Cross–sectional TEM image of encapsulated Al top gate. o) Transmission (*T*_r_) versus *V*_DS_ for the 10 nm transistor at a temperature of 225 K. Inset shows conduction band diagram for the carrier transport cross the barrier (left), *T*_r_, and effective velocity (*V*_EFF_) as a function of temperature. Reproduced with permission.^[^ ^109^ ^]^ Copyright 2016, IEEE.

Despite the first nanowire gate self‐aligned transistor demonstrated by Liao et al. in 2010^[^ ^97^ ^]^ (which has already been introduced in the previous part), a self‐aligned GaN nanowire gate transistor was also fabricated by Liao et al. with a minimum channel length of 45 nm.^[^ ^100^ ^]^ The fabrication flow can be seen in **Figure** **8**a. In specific, graphene is first mechanically peeled onto a SiO_2_/Si substrate, followed by the transfer of n‐type GaN nanowires onto graphene. Then, the source, drain, and gate electrodes are defined by lithography and metallization. A thin layer of platinum (Pt) is finally deposited over the whole structure and across the GaN gate. Unlike core‐shelled nanowires, in this transistor, the Schottky barrier with a clear rectification effect (Figure 8b) formed between GaN (gate) and graphene (channel) works as a semi‐high k gate dielectric (k≈10) to prevent leakage current and can be further modulated by changing the doping concentration in GaN, while GaN itself functions as the local gate. According to the transfer curve, this self‐aligned structure improves the gate's modulation of current from <10% to >50% (Figure 8c). A typical 100 nm channel length device can deliver a significant on‐current of 10 mA at *V*_DS_ = 1 V and *V*_TG_ = −0.5 V, a peak gm of 2.3 mS µm^−1^ at *V*_DS_ = 1 V, and a normalized current density of 2.3 × 10^9^ A cm^−2^ at *V*_DS_ = 1.8 V, which mainly attributes to the greatly reduced access resistance of the self‐aligned structure and the relatively short effective channel length. What's more, this 100 nm transistor shows a high intrinsic cut‐off frequency of up to 840 GHz, which is higher than Liao et al.’s previous work and mainly benefits from even fewer defects between directly connected GaN and graphene.

In 2011, Liao et al. further came up with a method to fabricate large‐scale self‐aligned nanowire gate transistor arrays.^[^ ^101^ ^]^ The corresponding fabrication flow is shown in Figure 8d. Monolayer graphene is first grown by CVD, then transferred onto a glass substrate, followed by patterning into blocks, external gate patterning, nanowire gate assembly, and a standard self‐aligned fabrication flow, which has been introduced previously. Note that in this work, both graphene and nanowire are fabricated in a large‐scale way (CVD and dielectrophoretic assembly, respectively). A typical 170 nm channel transistor shows robust electrical performances: an on‐current of ≈1.26 mA µm^−1^ at *V*_DS_ = 1 V and *V*_TG_ = 0.0 V, and a peak transconductance of 0.36 mS µm^−1^ can be obtained at *V*_DS_ = 1 V (V_g_ = 1.5 V). Though these transistors’ performance is limited by the poor quality of CVD compared to peeled graphene, the insulating glass substrate enables a small parasitic pad/gate capacitance ratio, which helps to achieve an extraordinarily high extrinsic cut‐off frequency of 55 GHz (Figure 8e) and allows to configure graphene transistor‐based radio‐frequency circuits. In experiment, a single graphene transistor‐based radio frequency doubler (Figure 8f) successfully works with the input signal frequency at 1.05 GHz and outputs most of the power concentrated at the doubling frequency of 2.1 GHz (Figure 8g).

In 2016, Yang et al. further adopted this self‐aligned method to fabricate sub‐100 nm transistors.^[^ ^102^ ^]^ Fabrication flow can be seen in Figure 8h; a schematic view and corresponding AFM image can be seen in Figure 8i. In this work, GaN nanowire also directly contacts the few‐layer MoS_2_ channel, forming a Schottky barrier (Figure 8j) between them with a turn‐on voltage of ≈3 V due to a p‐type GaN gate.^[^ ^103^, ^104^ ^]^ The 90 nm channel transistor fabricated in the experiment shows a current on–off ratio of 6 × 10^5^, an SS of 142 mV dec^−1^ at *V*_DS_ = 1 V, and a DIBL of 179 mV V^−1^ (Figure 8k), which is still smaller than previous works.^[^ ^105^, ^106^ ^]^ What's more, Yang et al. first calculated *D*_it_ (interface trap density) to determine the interface quality between the directly contacted GaN nanowire and the MoS_2_ channel. A value of 3.1 × 10^11^ cm^2^ eV^−1^ can be extracted for the device with a 230 nm channel length, which is one order of magnitude smaller than those reported^[^ ^107^, ^108^ ^]^ previously of directly depositing the uniform HfO_2_ dielectrics onto the MoS_2_ surface, further demonstrating high‐quality interface can be achieved by this gate transferring method. Drift velocity was also found to be constant at ≈1.4 × 10^6^ cm s^−1^ with varying channel length (Figure 8l), which is mainly influenced by phonon scattering due to a relatively clean interface.

In the meantime in 2016, a self‐aligned 10 nm channel transistor was successfully demonstrated by English et al.^[^ ^109^ ^]^ The fabrication flow can be seen in Figure 8m. The total device is fabricated on monolayer MoS_2_ grown by CVD on a Si/SiO_2_ substrate. At first, narrow external source/drain electrodes are patterned on MoS_2_, then a 2 nm seed layer of Al is deposited on MoS_2_ and is fully oxidized (Al_2_O_3_) in air. Next, thicker Al is deposited on Al_2_O_3_, while a 5 nm self‐passivated surface oxidation Al_2_O_3_ layer will be formed around the entire electrode, forming a final 10 nm‐length Al gate encapsulated by the Al_2_O_3_ dielectric (Figure 8n). Finally, a similar metal (Au) cross‐over deposition is done to finish the self‐aligned structure. In this method, a dielectric‐all‐around structure is used to separate the gate from deposited Au, which is different from several methods mentioned above, which mainly use the Schottky barrier to hinder carriers directly traveling from gate to source/drain electrodes. A fabricated 10 nm channel device exhibits high saturation current *I*_Dsat_ exceeding 400 µA µm^−1^ and SS of 250 mV dec^−1^. In addition, ballistic transport is also investigated in this work. The ballistic transmission efficiency (*T*_r_) of the transistor is ≈0.15 at *T* = 300–400 K in the saturation regime (Figure 8o), which gradually increases to 0.25 with an effective electron velocity (V_EFF_) of 9 × 10^5^ cm s^−1^ at *T* = 80 K, which mainly attributes to the suppression of the phonon scattering at a lower temperature.

#### 4.1.2 Recess Enabled Self‐Aligned Structure

**Figure 9:**
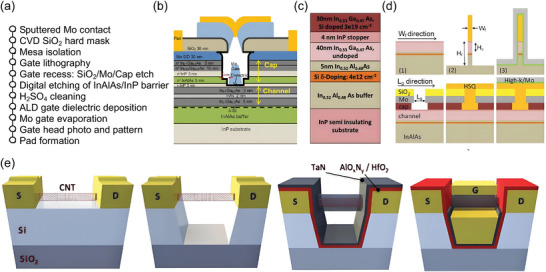
a) The very first fabrication flow which was designed in 2012. b) A detailed quantum well transistor, *L*_side_ is the distance between metal contacts and gate dielectric, which can be kept at 20–30 nm. Reproduced with permission.^[^ ^110^ ^]^ Copyright 2012, IEEE c) Starting heterostructure for FinFETs. d) Fabrication flow of III‐IV FinFETs, HSQ is kept to define fin shape. Reproduced with permission.^[^ ^111^ ^]^ Copyright 2016, IEEE e) Fabrication flow for self‐aligned nanowire GAA transistor. Reproduced with permission.^[^ ^112^ ^]^ Copyright 2012, IEEE.

The very first recessed gate self‐aligned MOSFET was achieved by Lin et al. in 2012 with sub‐30 nm channel length^[^ ^110^ ^]^ and its fabrication flow can be seen in **Figure** **9**a. This self‐aligned method is developed for quantum well‐based transistors, which make use of 2DEG (2D electron gas) trapped in potential wells originating from discontinuity of conduction and valence band in heterojunctions. The heterostructures in experiment can be seen in Figure 9b, the cap is used to reduce access resistance. To mention that, fabrication flow was gradually optimized by Lin et al. in 2012–2015 and the optimized fabrication process can be concluded as follows: i) Mo is first sputtered as metal contacts and SiO_2_ is deposited on Mo by CVD; ii) SiO_2_ mask and the W/Mo contact stack are etched by anisotropic RIE and etching will stop at the surface of n^+^ cap; iii) Mo is laterally undercut by isotropic RIE; iv) n^+^ cap is etched by anisotropic RIE and the etching will stop at few nanometers above the channel; v) DE (digital etching) is adopted to form final thickness of access region and channel. vi) thin film (e.g., 2.5 nm) HfO_2_ as a gate dielectric is conformally deposited and gate metal is then deposited by ALD. The gate length is determined by the recess process of SiO_2_, which can reach below 20 nm; due to the recess structure, the gate is precisely controlled next to drain/source electrodes while the length between the access region and gate dielectric can be kept at the range of 20–30 nm (*L*_side_, Figure 12b). At gate length of 30 nm, this 2DEG‐based MOSFET can achieve an extremely high transconductance of 1420 µS µm^−1^ and SS of 114 mV dec^−1^.

Recess method can also be used in III–IV FinFETs, which were successfully demonstrated in 2016 and achieved a 20 nm gate length.^[^ ^111^ ^]^ The starting heterostructure and corresponding fabrication flow can be seen in Figure 9c,d, respectively. After the deposition of Mo and SiO_2_, i) SiO_2_ mask and the Mo layers are etched by RIE; ii) *n* ^+^ cap is wet‐etched which results in a ≈20 nm undercut; iii) Fins are then patterned using HSQ by RIE and E‐beam lithography, to note that HSQ is kept in place in order to make modulation only is effective on sidewalls; iv) DE is adopted to further thin down the fin and smooth the sidewalls; iv) Al_2_O_3_ and HfO_2_ are deposited by ALD (EOT≈1 nm); v) Mo is sputtered as gate metal and patterned by RIE. The final gate length is also determined by recess of SiO_2_. During the experiment, FinFETs with *L*_g_ = 30 nm, *W*_f_ = 22 nm, and channel height of 40 nm exhibit an extremely high g_m_ of 1400 µS µm^−1^, an SS of 170 mV dec^−1^, and DIBL of 220 mV V^−1^. Besides, the self‐aligned structure enables a *R*_on_ at 180 Ω·µm.

A recess of Si substrate to suspend CNT channel to form a self‐aligned GAA device was successfully demonstrated by Franklin et al.^[^ ^112^ ^]^ The critical part lies in a wet etch method which is highly selective to <100> Si substrate and will not damage CNT channel, which finally leads to a self‐aligned GAA structure. The main fabrication process can be concluded as follows (Figure 9e): i) CNTs are transferred on SOI with a <100> Si; ii) electron beam lithography and metal deposition/lift‐off are used to define Pd source/drain contacts; iii) wet etch is used to etch the silicon beneath CNTs, while the Si beneath source/drain electrodes won't be etched due to protection of S/D. After etching, the Si substrate is recessed and CNTs are suspended; iv) ALD is adopted across the whole device in order to coat CNTs with oxide and gate metal (TaN); v) a final Pd gate electrode is established to contact the TaN; vi) The deposited TaN on S/D can be selectively removed during lift‐off because of the trench beneath channel; vii) unwrapped TaN will be removed by RIE, while the left Pd/TaN forms the final gate. The spacers between GAA and S/D electrodes are the same as dielectric thickness, which shows a self‐aligned feature. This self‐aligned GAA transistor can avoid defects from supporting oxide in typical CNTs devices, owns a 30 nm channel length with a high on‐current of ≈16 µA at V_gs_–*V*_th_ = 1 V with a peak transconductance of ≈45 µS and an inverse SS of ≈105 mV dec^−1^.

### 4.2 Nanogap Transistor

Nanogap, which means a pair of metals or semiconductors with a nanometer scale distance, has developed a lot and is gaining attraction to build nano‐scale electronic devices. For transistors, nanogap can enable an ultra‐short channel length. Therefore, using nanogap‐related method to determine the channel length has great potential. Nevertheless, though there are lots of developed methods, such as photolithography, electron‐beam lithography (EBL), Focused Ion Beam milling (FIB), and some other methods to fabricate nanogap and have already been concluded in previous reviews,^[^ ^113^, ^114^, ^115^ ^]^ the fabrication of nanogap in transistors still needs more works. The following part will introduce some achievements in nanogap transistors using different methods.

#### 4.2.1 Direct Nanogap Fabrication Between Electrodes

**Figure 10:**
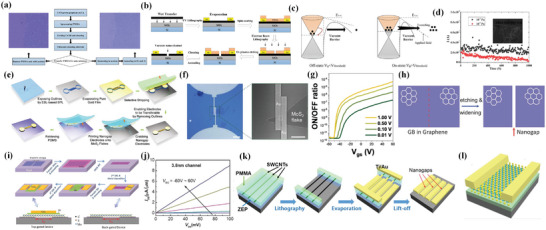
a) Optimized wet transfer method for graphene; b) Fabrication flow of vacuum channel transistor. c) Band structure of vacuum channel transistor at off and on state. d) Stability test for vacuum channel transistor. Reproduced under the terms of the CC‐BY license.^[^ ^116^ ^]^ Copyright 2018, The Author(s). e) Fabrication flow of SPL+transfer printing. f) transistor fabricated by SPL+transfer printing. g) Transfer characteristics of SPL transistor. Reproduced with permission.^[^ ^120^ ^]^ Copyright 2020, WILEY‐VCH Verlag GmbH & Co. KGaA, Weinheim. h) Schematic view of grain boundary widening etching by H_2_ plasma. i) Fabrication flow of top and back gate transistors based on graphene nanogap. j) Transfer characteristic for 3.8 nm channel transistor, showing a nearly Ohmic contact between channel (MoS_2_) and graphene electrodes. Reproduced with permission.^[^ ^122^ ^]^ Copyright 2017, WILEY‐VCH Verlag GmbH & Co. KGaA, Weinheim. k) Fabrication flow for large‐scale nanogap by adopting SWCNT as a mask. l) Schematic view of the 7.5 nm channel transistor. Reproduced with permission.^[^ ^123^ ^]^ Copyright 2019, American Chemical Society.

In 2019, Xu et al. fabricated a 90 nm vacuum channel transistor, which allows electrons to travel through the nanoscale vacuum channel.^[^ ^116^ ^]^ In this work, graphene is first grown on Cu by CVD, followed by an optimized wet transfer method including ultrasound cleaning and post‐thermal annealing (**Figure** **10**a) to enhance graphene's quality on a Si/SiO_2_ substrate. After transferring, the transistor's fabrication flow can be seen in Figure 10b. 90 nm vacuum channel is determined by the standard EBL process. The transistor's operation relies on electron tunneling from the graphene emitter to the vacuum; that is, by combing the back gate modulation on graphene's Dirac point and bias voltage modulation on the vacuum barrier (Figure 10c), electrons can be emitted from the emitter and collected from the collector. This transistor shows an on–off current ratio up to 10^2^ with low working voltages (<20 V) and low leakage current (<0.5 nA), attributes of thick SiO_2_. What's more, this transistor has a desirable performance in the stability test at 10^−4^ Pa (Figure 10d) due to the high thermal conductivity of graphene, which has not been demonstrated in previous works.

To note that, though EBL is a promising tool for nanoscale fabrication, it still has certain intrinsic drawbacks: i) It's extremely time‐consuming. In common cases, exposure time can exceed 24 h per square centimeter.^[^ ^117^ ^]^ ii) EBL process is not suitable for lots of substrates or functional devices.^[^ ^118^, ^119^ ^]^ In 2020, Duan et al. adopted SPL (sketch and peel lithography) and transfer printing to solve the drawbacks mentioned above.^[^ ^120^ ^]^ Specifically (Figure 10e), SPL is conducted on the donor substrate, only outlines of two electrodes are needed to be etched by EBL, so the total exposure time is greatly reduced. Then, by depositing electrode metal, removing extra metal outside of closed outline, and removing resist, a pair of electrodes with nanogap can be made. Then, this pair of electrodes will be transferred from donor to acceptor substrate by a polyfilm‐assisted transfer method. This SPL and transfer printing method significantly reduces the exposure time and the proximity effect of EBL for defining the multiscale electrodes because only single‐pixel outlines need to be exposed. In this work, a MoS_2_ transistor with 70 nm channel length was fabricated through this method (Figure 10f). It showed an on–off ratio of ≈10^7^ (Figure 10g), which demonstrates the feasibility of this SPL+ transfer printing method.

The former two nanogap transistors are still limited by the resolution of EBL, while the following part will introduce several nanogap transistors based on other advanced methods.

Xie et al. found a novel grain boundary widening H_2_ etching technique in graphene basal plane.^[^ ^121^ ^]^ In 2017, they further adopted this method to fabricate a sub‐10 nm channel‐length transistor.^[^ ^122^ ^]^ The etching mechanism can be simply concluded as, C─C bond configurations in graphene can enable anisotropy etching along the grain boundary, while H‐radicals attack the carbon atoms at both edges and surface defects via C─C bond breakage (volatilization) and C─H covalent bond formation (hydrogenation). Since methane is the main reaction product, the grain boundaries will be broadened after hydrogen plasma etching, forming a zigzag‐shaped nanogap (Figure 10h), and the gap width can be well controlled by controlling temperature and plasma dose. After etching nanogaps by H_2_, the channel material (MoS_2_) is then transferred onto the nanogap, while the remaining graphene serves as the source‐drain electrodes (Figure 10i). The monolayer graphene forms Ohmic contact with MoS_2_ (Figure 10j) in the 3.8 nm nanogap channel transistor, exhibiting a current on–off ratio of ≈2.6 × 10^6^, off‐state current density of ≈5 pA mm^−1^, SS of 208 mV dec^−1^, and a relatively large DIBL of ≈1.03 V V^−1^, which can still satisfy the ITRS requirements.

In 2019, Xiao et al. demonstrated a large‐scale nanogap fabrication method by making use of CNT as a mask.^[^ ^123^ ^]^ In specific, horizontally‐aligned SWCNTs grown by CVD will be transferred on ZEP‐520A resist with the assistance of PMMA, thus forming a ZEP/SWCNTs/PMMA sandwich structure. Then, EBL will be adopted to define parallel trenches perpendicular to SWCNTs. As a result, a portion of the SWCNT is suspended on the substrate, whereas the rest is till sandwiched between the ZEP and PMMA. The suspended SWCNTs will work as masks in following metal evaporation and lift‐off process, thus nanogaps between deposited metals will be formed under SWCNTs (Figure 10k). Since the length of nanogap is determined by the diameter of SWCNT, it is suitable for fabricating large‐scale, uniform channel length transistors. In experiment, a series of 7.5 nm (MoS_2_) channel length back gate transistors were successfully fabricated by evaporating using SWCNTs as masks on MoS_2_ thin film, then transferring both MoS_2_ and evaporated electrodes to HfO_2_/Si substrate (Figure 10l). A typical as‐fabricated transistor shows mobility of 17.4 cm^2^ V^−1^s^−1^, SS of 120 mV dec^−1^, and an on–off ratio of ≈10^7^.

#### 4.2.2 Nanogap Fabrication for Dielectric

Aside from directly patterning nanogap between electrodes, fabricating nanogap on dielectric is also a potential way to fabricate short‐channel length devices. The following part will introduce two works that are relevant to this method.

**Figure 11:**
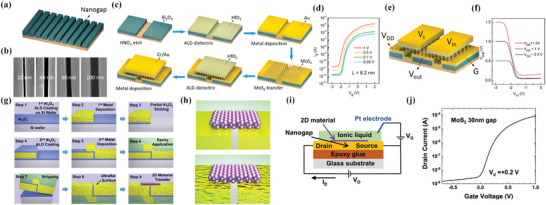
a) Schematic view of parallel nanogaps on Bi_2_O_3_. b) Different nanogap widths can be achieved by controlling corrosion time. c) Fabrication flow of sub‐10 nm transistor based on Bi_2_O_3_ nanogap. d) Transfer characteristic of 8.2 nm channel transistor. e) Schematic view of the inverter fabricated based on Bi_2_O_3_ nanogap. f) VTC of as‐fabricated inverter. Reproduced with permission.^[^ ^124^ ^]^ Copyright 2017, American Chemical Society. g) Fabrication flow of sub‐10 nm nanogap caused by Al_2_O_3_. h) Comparision between ultraflat surfaces originates from the stripping process and deposited metal surfaces. i) Schematic view of 30 nm channel transistor. j) Transfer characteristic of 30 nm MoS_2_ channel transistor. Reproduced with permission.^[^ ^125^ ^]^ Copyright 2021, American Chemical Society.

In 2017, Xu et al. made use of corrosion cracking along with the cleavage plane to large‐scale fabricate nanogap on bismuth trioxide (*β*‐Bi_2_O_3_).^[^ ^124^ ^]^ In the short term, after depositing Bi_2_O_3_ on Si and the annealing process, stress will develop due to the different expansion coefficients between Bi_2_O_3_ and Si. Then, annealed Bi_2_O_3_ will be immersed in HNO_3_. The combined effects of released stress and acid corrosion will cause steep, uniform, and parallel nanogaps along every crystal domain's cleavage plane (**Figure** **11**a). The width of nanogaps can be defined from several to hundreds of nanometers with different immersive times (Figure 11b). In the experiment, ultrashort channel devices were fabricated on the *β*‐Bi_2_O_3_ sub‐10 nm nanogaps (Figure 11c). The fabrication flow determines that the width of the nanogap can be further decreased by dielectric deposition, and the final channel length, as well as the device performance, can be largely influenced by the thickness and quality of the deposited dielectric. In the experiment, a MoS_2_ channel transistor with a channel length of 8.2 nm exhibited promising electrical transport performance, including a low SS of 140–170 mV dec^−1^, a high current density of 2.5 µA µm^−1^, and a high current on–off ratio of ≈10^7^ (Figure 11d). Also, an inverter was built using this method (Figure 11e), showing a good VTC (voltage transfer curve) and a relatively high voltage gain (the maximum gain is above 2 V V^−1^) (Figure 11f).

In 2021, Namgung et al. also demonstrated a way to fabricate sub‐10 nm nanogap, in which the gap width is determined by the thickness of Al_2_O_3_ deposited by ALD.^[^ ^125^ ^]^ The overall fabrication flow can be seen in Figure 11g. As the fabrication flow shows, since the final structure comes from stripping and a flipping process, two separated metal parts together with the middle Al_2_O_3_ have an ultra‐flat surface due to the flatness of the Si wafer and atomically controlled layer‐by‐layer deposition of Al_2_O_3_ (Figure 11). A maximum root‐mean‐square (RMS) roughness for template‐stripped Au can still reach down to 0.350 nm, while the RMS roughness of the as‐deposited Au surface is 1.177 nm by AFM. This difference will theoretically enable a lower contact resistance when 2D material is then transferred onto this flat surface compared to its deposited metal counterpart. A short‐channel transistor with a 30 nm gap has been fabricated during the experiment (Figure 11i). Two metal parts work as source and drain electrodes; the deposited MoS_2_ works as the channel; and an ionic liquid works as the gate electrode. This transistor shows an on‐off ratio of ≈10^4^ (Figure 11j), and the SS value is measured as low as 83 mV dec^−1^.

It's worth noting that, nanogap dielectric might influence the transistor's performance, such as SS and leakage current, especially when nanogap dielectric directly contacts the channel (though neither of these two works has a directly contacted dielectric substrate and channel, in,^[^ ^125^ ^]^ Al_2_O_3_ in all samples is well below the two electrodes by AFM image for some reasons). Under this scenario, interface traps will impact carriers' mobility via Coulomb scattering. It's important to carefully control the surface roughness in layer‐by‐layer deposition flow and defects on the dielectric substrate's surface.

#### 4.2.3 Other Relevant Methods to Fabricate sub‐10 nm Nanogap Transistor

**Figure 12:**
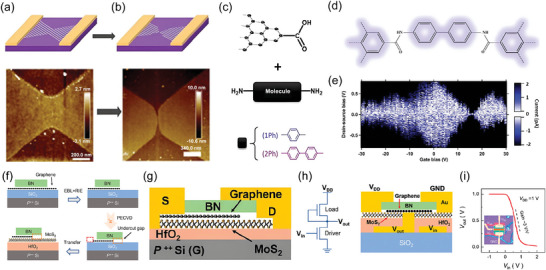
a) A bowtie‐shaped graphene channel after etching. b) Electroburning enabled nanogap. c) Either 1,4‐diaminobenzene (1Ph) or 4,4′‐diaminobiphenyl (2Ph) is used to reconnect two graphene electrodes. d) Discrete conjugation means discrete electron energy, which forms an electrode‐barrier‐conductive island‐barrier‐electrode single electron transistor structure. e) Demonstrating for single electron transport mechanism. Reproduced with permission.^[^ ^126^ ^]^ Copyright 2017, American Chemical Society. f) Fabrication flow of hydrogen selectively etch enabled undercut structure. g) Schematic view of undercut nanogap‐based transistor. h) Schematic view of as fabricated inverter. i) VTC of as fabricated inverter. Reproduced with permission.^[^ ^127^ ^]^ Copyright 2023, American Chemical Society.

In 2017, Xu et al. demonstrated a robust way to controllably introduce a sub‐1 nm gap in graphene by electroburning with a successful ratio of 88%.^[^ ^126^ ^]^ In specific, a graphene channel transistor is first fabricated, and then lithography is adopted to decrease the channel width in the middle channel, forming a bowtie shape (**Figure** **12**a), which is essential for a highly successful ratio of nanogap fabrication. Then, a feedback loop is connected to the transistor, causing it to be electroblasted by joule heat. Once the circuit detects a negative differential conductance, the loop will shut down, and the graphene can be precisely etched (Figure 12b). Since the graphene is etched in air, the Joule‐heat‐induced oxidation process forms carboxylic acid groups (−COOH) on the edges of the etched graphene. This natural merit is further used to fabricate a single‐electron transistor by reconnecting separated graphene with either 1,4‐diaminobenzene (0.6 nm) or 4,4′‐diaminobiphenyl (1 nm) (Figure 12c). The break of electronic conjugation from benzene ring to amide (Figure 12d) at molecular contacts suggests the confinement of discrete molecular orbitals and results in discrete electron energy, which exactly matches the structure requirement of a single electron transistor. The later electrical test successfully shows Coulomb blockade diamonds (Figure 12e), which is a demonstration of single electron transfer.

In 2023, Tian et al. further utilized the H_2_ selective etching for graphene to fabricate sub‐10 nm transistors.^[^ ^127^ ^]^ The fabrication flow can be seen in Figure 12f and the final transistor structure can be seen in Figure 12g. During fabrication, edge‐aligned graphene/BN heterostructures are achieved by EBL and RIE, while the undercut etching on graphene is achieved by H_2_ plasma etching demonstrated by Xie et al.^[^ ^116^ ^]^ This undercut length can be well controlled under 10 nm and determines the final channel length. The fabricated 9 nm channel length transistor shows a high on–off current ratio of 3 × 10^7^ at *V*_DS_ = 1 V and exhibits excellent SS of 120 mV V^−1^ at *V*_DS_ = 1 V with an average room temperature mobility of 72 cm^2^ V^−1^ s^−1^. The excellent performances of this 9 nm transistor are mainly attributed to the strongly suppressed electron scattering in the transferred BN self‐encapsulating structure and the high‐quality, single‐crystalline, monolayer MoS_2_ channel grown by epitaxial technique. An inverter is also demonstrated by adopting a similar fabrication method (Figure 12h). This inverter also has an ideal VTC (nearly full voltage swing) and a maximum voltage gain at 3 V V^−1^ (Figure 12i), which is larger than previous Xu et al.’s work.^[^ ^124^ ^]^

## 5 Design of Small Feature‐size Transistors for Reduced Footprint

Although the smaller gate and channel length as discussed above contributed to a significantly reduced feature size of transistors, what counts and establishes the level of integration in a single chip is the transistor's footprint. Generally, the footprint of a transistor can be defined by the formula: footprint = gate length + 2 × contact length.^[^ ^128^ ^]^ It is evident that to obtain a compact footprint for typical transistors, both the gate length and the contact length must be reduced. Therefore, we would like to further discuss the advantages of several small feature transistors based on low‐dimensional material for achieving a reduced footprint in this section, where the contact length scaling method, device structure design, and functional integration toward a small footprint would be introduced. And finally, several cutting‐edge applications of small‐footprint transistors based on low‐dimensional materials were proposed to prove their practical possibility in the post‐Moore era.

### 5.1 Contact Size Scaling

**Figure 13:**
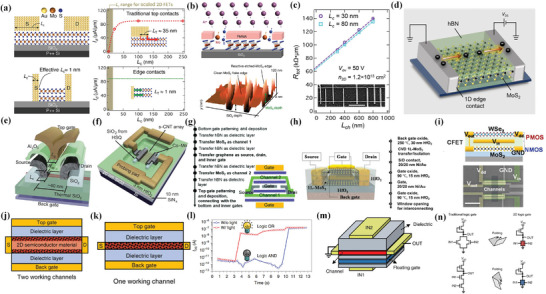
a) Schematic of a bilayer 2D FET with traditional top contacts, on‐current diminishes as the top contact length decreases (top); Schematic of a bilayer 2D FET with edge contacts and an effective *L*_c_ ≈ 1 nm, leading to the possibility of on‐current that is independent of contact length (bottom). b) Schematic of the etch process with only contact regions selectively bombarded by Ar^+^ ion beam (top); 3D AFM image highlights the reactive etched MoS_2_ edges and the relatively clean MoS_2_ flake edges. c) Edge contact resistance with different contact length on the same 2L MoS_2_ film. Reproduced with permission.^[^ ^133^ ^]^ Copyright 2019, American Chemical Society. d) Schematic illustration of the transistor. Reproduced with permission.^[^ ^128^ ^]^ Copyright 2019, American Chemical Society. e) Schematic view showing the oxide trench defining the 40‐nm device footprint, and the end‐bonded source/drain contacts to the s‐CNT channel. *L*_c_, *W*_sp_, and *L*_g_ mark the device contact length, spacer width, and gate length, respectively. f) Schematic showing a s‐CNT‐array transistor scaled to a 40‐nm‐device footprint with the channel sitting on a 3‐nm HfO_2_/Si back gate and the probing pads on 10‐nm SiN_x_/20 nm SiO_2_ field oxide. Reproduced with permission.^[^ ^134^ ^]^ Copyright 2017, The American Association for the Advancement of Science. g) Key fabrication flow of the MBCFET fabricated by Huang et al. Reproduced with permission.^[^ ^135^ ^]^ Copyright 2020, IEEE. h) Schematic view of the MBCFET with its corresponding fabrication flow fabricated by Cao et al. i) Structure schematic and SEM image (scale bar: 5 µm) of the MoS_2_/WSe_2_ CFET inverter. Reproduced with permission.^[^ ^137^ ^]^ Copyright 2021, IEEE. j) Thick 2D transistor with two working channels. k) Thin 2D transistor with one working channel. l) The conversion from AND logic to OR logic after light adsorption. m) Schematic view of 2D floating‐gate transistor. n) Reduced area for 2D transistor. Reproduced with permission.^[^ ^140^ ^]^ Copyright 2019, The Author(s), under exclusive license to Springer Nature Limited.

Though channel length and gate length scaling for 2D material have been studied, contact length scaling is still lacking in exploration. A practical issue is that, as the contact length is scaled close to or even under the carrier transfer length (the length over which the majority of carriers are injected) in a conduction channel, the area available for carrier injection is reduced, which dramatically increases the contact resistance owing to the current crowding. Therefore, to further accomplish small‐footprint transistors with great electrical performance, new approaches must be found to simultaneously achieve lower contact resistance and a smaller contact length. Several contact engineering methods have already been put forward for 2D FETs, such as interface engineering and using different materials.^[^ ^129^, ^130^, ^131^ ^]^ However, all these methods still need to sacrifice contact length (hundreds of nanometers) to achieve small contact resistance. Then, contacting metal and channel by edge contact has drawn attention, since the injection area at the edge is independent of the physical contact length and the edge contact geometry is especially suitable for 2D materials due to their atomically controllable thickness. According to the ideal theoretical model of edge contact, metal electrodes will be directly bonded to the side of the semiconducting channel and carriers will transfer from electrodes to channel via covalent bonds. This configuration enables I_D_ to keep as a constant even when the contact length is greatly scaled (**Figure** **13**a). However, the demonstration of edge contact between metal and 2D material used to be limited to the use of an ex situ and isotropic plasma etching approach^[^ ^132^ ^]^ before 2016. But in this part, we will introduce an optimized edge contact.

In 2019, Cheng et al. first put forward an edge‐contacted MoS_2_ FETs by using an in situ Ar ion beam etching method.^[^ ^133^ ^]^ Briefly speaking, in situ edge contact is realized by first adopting ion beam lithography to etch the 2D channel's contact region, followed by depositing metal in the same chamber to finish edge contact. The schematic view of ion beam etching on MoS_2_ can be seen in Figure 13b. The generated active edge after etching can react with the metal, forming a bonded edge interface. This etching method can be quite clean and won't change the properties of the inside channel. By adopting the in situ etching method, FETs with contact lengths ranging from 20 to 600 nm (channel lengths all equal to 600 nm) showed uniform SS and transfer curves. What's more, as contact length decreases from 80 to 20 nm, the resistance increases only ≈30%, from 950 to 1230 Ω. The total contact resistance is still negligible to the total resistance of the device (Figure 13c). It's worth noting that, this in situ edge contact also exhibits an order of magnitude higher performance compared to the best‐reported ex situ metal edge contacts.

Similarly, in 2019, Jain et al. reported a viable edge contact formation to hBN‐encapsulated monolayer MoS_2_ in a polymer‐free manner.^[^ ^128^ ^]^ By combining the techniques of reactive ion etching, in situ Ar^+^ sputtering at a slightly tilted angle to reduce contact resistance and annealing, the schematic view of a typical as‐fabricated transistor can be seen in Figure 13d. The transistor has a channel length of 1000 nm and contact length of 500 nm, exhibiting relatively high mobility up to 30 cm^2^ V^−1^ s^−1^, high on‐current density >50 µA µm^−1^, and low SS of 116 mV dec^−1^ are achieved. Also, the contact resistance can be deduced to be *R*_C_·*W* = 27.8, 11.7, and 8.3 kΩ·µm at *V*_DS_ = 1, 2, and 3 V, respectively, which is still acceptable.

Though the two papers we list here did not design transistors with both short channel lengths and small contact lengths simultaneously, nevertheless, edge contact based on the in situ ion beam etching provides a possible approach in further scaling contact lengths thus achieving a small footprint for those small channel devices. What's more, aside from edge contact, heterophase engineering is also a promising way to achieve small contact sizes with low resistance, two transistors, especially one that adopts CNT as gate, fabricated by this method, have already been introduced in Section 3.1.

### 5.2 Transistor Structure Design in Achieving Small Footprint

Aside from contact size scaling, which provides a possibility to further scale down the transistor's footprint, this part will introduce several transistor structure design methods that have contributed to the small footprint or reduced area compared to current circuit formation.

In 2017, Cao et al. demonstrated a method to fabricate array transistors with uniform footprints of 40 nm, and channel lengths ranging from 11 to 50 nm.^[^ ^134^ ^]^ The schematic view of a single transistor can be seen in Figure 13e. To determine the overall footprint of this transistor, as‐grown P‐type CNT arrays are first transferred to Si/SiO_2_ substrate. Then, hydrogen silsesquioxane (HSQ) is spun onto the CNT arrays and patterned into bar shapes by EBL. This step determined the overall footprint (≈40 nm, which is the distance between two bars) of the transistor. HSQ bars were then transferred into SiO_2_ bars by annealing at 650°C. To fabricate a single transistor, since bars cover the CNT array, extra CNT between two bars will be etched away. Therefore, only one CNT will remain as a channel between two bars, while array devices’ fabrication doesn't need this step. To overcome high contact resistance at a limited overall footprint, Cao et al. adopted a low‐resistance end‐bonded contact technique by making use of carbon dissolution between CNT channel and metal electrode The following steps are gate dielectric deposition and gate metal definition for individual transistor, while the array devices adopt bottom gate structure (Figure 13f). A typical individual transistor possesses an overall footprint of 40 nm, an 11.2 nm channel length, 10.7 nm, and 8.8 nm for source contact length and drain contact length, respectively. 11.2 nm channel length device shows an SS at 85 mV dec^−1^, which means its immunity to SCEs, an Ion of 2 µA at *V*_DS_ = −0.5 V, V_gS_ = −0.5 V, which demonstrates a low contact resistance formed by end‐bonded contact. Also, the s‐CNT array devices were tested, though these array devices suffer from large variations of SS (large SS, ≈500 mV dec^−1^, *I*_off_ = 20 µA µm^−1^ at *V*_OV_ = −0.2 V) and *V*_T_ due to randomly distributed fixed charges in gate oxide, they still exhibit a high‐saturation on‐state current above 1.2 mA µm^−1^ and conductance above 2 mS µm^−1^, which exceeds that of the best‐competing silicon devices when they are benchmarked under the same gate overdrive and *V*_DS_ without any normalization.

The multi‐bridge channel field‐effect transistor (MBCFET), which is fabricated by vertically stacking the conductive channels in a gate‐all‐around configuration, has been identified as a possible method to achieve ultimate transistor scaling because of its exceptional electrostatic controllability and power/area efficiency. The very first MBCFET using MoS_2_ as channel was achieved by Huang et al^[^ ^135^ ^]^ in 2020. A 2‐level‐stacked MoS_2_ ultrathin MBCFET with channel thickness of 0.6 and 1.2 nm, channel length over 28 nm, hBN dielectric layer, graphene electrodes, and a GAA geometry were successfully demonstrated. Cross section of the device and corresponding key fabrication process can be seen in Figure 13g. The vdW heterostructures were adopted, which guaranteed the clean interface without contamination, enabling high‐efficient carrier transport and good gate controllability: a high normalized drive current of 13.2 mA µm µm^−1^ at *V*_DS_ = 1 V, and a minimum theoretical value SS of 60 mV dec^−1^ at room temperature. What's more, the leakage current of the device is as low as 0.92 pA µm^−1^ due to the large bandgap in thin MoS_2_ film channel, which is only 6.5% of that in the Si MBCFET. Huang et al. further explored the operation mechanism of MoS_2_ MBCFET in 2021 by adopting a similar structure but both channel thickness equal to 2 nm and channel length above 20 nm.^[^ ^136^ ^]^ However, the research on these two transistors remained on the device itself. Nevertheless, a MBSFET has directly explored its potential to reduce footprint fabricated by Xiong et al. in 2021^[^ ^137^ ^]^ (Figure 13h). Instead of using mechanically exfoliated MoS_2_ flakes, Xiong et al. used CVD to synthesize large‐scale monolayer MoS_2_ to demonstrate MBCFET in large‐scale fabrication. During the experiment, MBCFET with various channel lengths (from 160 nm to 2 µm) with gate lengths of 100 nm were tested. Robust electrical transport performance including low SS of 126 mV dec^−1^, high on–off ratio of 10^7^, and large on‐state current density of 420 mA µm^−1^ at *V*_DS_ = 1 V with a low contact resistance of 0.77 kΩ µm per channel footprint were successfully demonstrated. Furthermore, the MoS_2_/WSe_2_ CFET device fabricated by a monolithic 3D stacking method based on CVD‐grown materials for the first time was demonstrated. A schematic view of this device can be seen in Figure 13i. The fabrication process of the CFETs is similar to the previous MBCFET but replaces the second n‐type MoS_2_ channel with a p‐type channel WSe_2_, thus forming an inverter (including both NMOS and PMOS in one transistor) with a maximum gain of ≈9 V V^−1^ at *V*_dd_ = 3 V. Since two channels of CFET share one gate, the overall occupied area (or, the footprint) is halved. Last but not least, for MBCFETs, there are still several possible methods to further scale the whole device, such as shorter channel length realized by a widening technique to fabricate shared graphene electrodes^[^ ^138^ ^]^ and a technique to grow uniform/ultrathin high k dielectrics with an equivalent oxide thickness of 1 nm.^[^ ^139^ ^]^

### 5.3 Functional Integration in a Single Device

Multifunctional device, which means one transistor can realize several functions that used to need more transistors, combines the advantages of low dimensional material in scaling and can also achieve power/area efficiency and high integration. Though a multifunctional design has already been introduced in this review (multi‐CNT gates transistor), this part will additionally introduce one more multifunctional device trying to achieve a reduced footprint.

In 2019, Liu et al. demonstrated a double gate transistor, which can achieve photo‐switching logic (switching between OR and AND logic).^[^ ^140^ ^]^ Liu et al. first adopted a transistor design with a top gate and a bottom gate, MoS_2_ was adopted as the channel with various thicknesses, hBN and HfO_2_ were chosen as the top dielectric and back dielectric layers, respectively. This single transistor can achieve OR logic when the channel is thicker than 4 nm and can achieve photo‐switching logic between AND and OR logic when the channel is thinner than 4 nm. The operation mechanism is that when the channel is thick enough (Figure 13j), both the top and bottom gates can control an individual surface channel. So only one positive input is required to turn on the device but two negative inputs are needed to turn off the device, which corresponds to OR logic. When the channel length is thinner than 4 nm (Figure 13k), the overall channel thickness is thinner than the screening length in MoS_2_, so both gates can individually turn on or turn off the device, which is the AND logic. For thin channel devices, both OR and AND logic can be achieved when light is applied. After adsorbing light, extra electrons will be lifted from the valence band to the conductance band, thus individual gates can no longer turn off a thin channel device, leading to logic switching performance from AND to OR (Figure 13l). Moreover, after inserting an additional graphene layer into the back‐gate dielectric as the trap layer (Figure 13m), this new 2D floating‐gate transistor was found to be able to realize in situ memory. State‐0 can be stored in this transistor by a subsequent write‐0 (positive back gate voltage pulse is applied) operation, state‐1 can be stored in the device by a subsequent write‐1 (negative back gate voltage pulse is applied) operation. To mention that, though no clear footprint has been stated, this transistor achieves a 50% area reduction in building OR logic and AND logic circuits (Figure 13n). This area reduction is attributed to the special double‐channel modulation in a single transistor, which means only 1 transistor instead of 2 is needed to build the pull‐down circuit.

#### 5.3.1 Advanced Applications Enabled by Small Footprint Transistor

Despite the possible scaling size achieved by transistors, it is also important to find out their practical applications. Though several possible applications, such as inverter and different logic gates have been introduced in this review, this part will additionally introduce several cutting‐edge applications based on 2D transistors.

#### 5.3.2 Logic‐in‐Memory Computing

Nowadays, integrated circuits are facing severe challenges caused by data‐intensive applications. In short terms, traditional von Neumann computing architecture, where processing center and memory units are physically separated, is facing bottlenecks due to the larger time consumption of data transmission between processing and memory units compared to computing itself. This bottleneck can be very tough for Si‐based transistors to solve since Si has its limitations in ultimate scaling (SCEs). However, 2D material has the potential to integrate both multiple logic computing and in situ memory by either adopting a floating gate^[^ ^140^, ^141^ ^]^ or ferroelectric^[^ ^142^ ^]^ in one transistor, which provides a possible solution for the von Neumann limitation.

Typically, to achieve memory function, a floating gate mainly makes use of the tunneling effect. Take Liu et al.’s double gate transistor structure^[^ ^140^ ^]^ as an example, in which the floating gate is inserted between the bottom gate and the channel, isolated by a dielectric. A large positive (negative) voltage pulse applied on the bottom gate induces (electrons) holes in the channel and leads to the electrons (holes) tunneling into the floating gate. Once the pulse is diminished, electrons (holes) will be trapped in a floating gate. This process corresponds to memory. Numerically speaking, a voltage pulse should be large enough to help carriers tunnel through the dielectric and make sure the floating gate is sufficiently charged, so the effect of the sufficient stored electrons (holes) can resemble the behaviors that a negative (positive) voltage is applied on floating gate, which demonstrates that the input signal on floating gate before voltage pulse can be successfully memorized.

**Figure 14:**
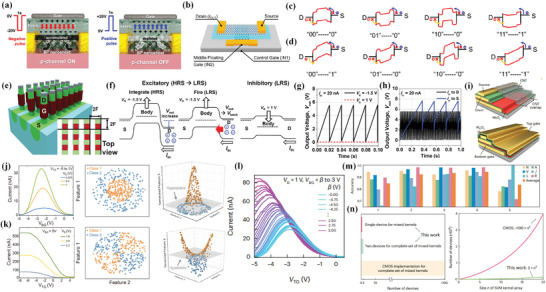
a) Schematic view of a ferroelectric p‐type FET. Reproduced with permission.^[^ ^142^ ^]^ Copyright 2015, American Chemical Society. b) Schematic view of the logic‐in‐memory transistor. c) Band diagram of AND logic. Reproduced with permission.^[^ ^143^ ^]^ Copyright 2023, American Chemical Society. d) Band diagram of XNOR logic. e) Schematic view of vertical GAA synaptic transistor. f) Band diagram of the synaptic transistor at excitatory function (left) and inhibitory function (right). g) Spiking characteristic at excitatory function (black line) and inhibitory function (red dashed line). h) Schematic view of the MBCFET with its corresponding fabrication flow fabricated by Cao et al. Reproduced with permission.^[^ ^147^ ^]^ Copyright 2021, Wiley‐VCH GmbH. i) Structure schematic of MKH transistor. j) Three Gaussian kernel functions generated by MKH transistor (left), two classes of randomly distributed data with a concentric distribution shape (middle), classification by Gaussian kernel (right). k) Three sigmoid kernel functions generated by MKH transistor (left), two classes of randomly distributed data in a diagonal way (middle), classification by sigmoid kernel (right). l) Mixed kernel characteristic. m) Classification accuracy for different mixed kernels corresponding to back gate voltage values of −5, −3, −1, 1, and 3 V. Mixed‐kernel type 1 is purely Gaussian, mixed‐kernel type 5 is purely sigmoid, and mixed‐kernel types 2–4 are 75%/25%, 50%/50%, and 25%/75% Gaussian/sigmoid kernels, respectively. n) Comparison of the number of devices needed to generate a complete set of mixed kernels for CMOS and MKH implementations (left), Comparison of the number of devices needed to implement an *n* × *n*‐kernel matrix for CMOS and MKH implementations (right). Reproduced with permission.^[^ ^151^ ^]^ Copyright 2023, The Author(s), under exclusive license to Springer Nature Limited.

While for the transistor which adopts ferroelectric (**Figure** **14**a), mainly relies on the coercive voltage caused by ferroelectric to memorize the previous logic state. When the voltage pulse applied on the control gate is higher than the coercive voltage (which is typically much larger than the voltage needed in the program state) applied to ferroelectric, ferroelectric dipoles could be aligned (electrical polarization), forming an opposite electric field compared to a voltage pulse. Taking a p‐type transistor as an example.^[^ ^142^ ^]^ When the transistor is in on (off)‐state, holes (electrons) are induced in the channel, and a large negative (positive) pulse on the control gate can cause a positive (negative) electric field in ferroelectric thus maintaining holes (electrons) accumulation even when no voltage is applied on control gate later, thus successfully memorizing the previous logic state.

Combining the intrinsic advantage of 2D material to achieve multiple logic functions in one transistor, it is possible to further integrate logic‐in‐memory devices. In this part, we will introduce a more recently designed transistor demonstrated by Sheng et al.,^[^ ^143^ ^]^ in which logic switching only relies on the amplitude change of voltage bias between drain and source electrodes. The schematic view of this transistor can be seen in Figure 14b. In this device, two gates (floating gate and bottom gate) serve as two input terminals, while the current in the channel is regarded as an output signal. This transistor can achieve both AND and XNOR logic by simply changing *V*_DS_. In specific, logic switching is achieved by modulating the polarity behavior of WS_2_ (channel). i) When *V*_DS_ is small enough, WS_2_ shows an n‐type transfer curve, which means a Schottky barrier between the channel and the drain electrode is always thick enough to hinder holes’ transport, only when two inputs are set to logic 1 (4 V) can the two‐channel barriers (one is controlled by the middle floating gate in the middle region of the channel, one is controlled by the bottom gate in the two terminals of the channel) be low enough to allow electrons’ transport (output logic 1), which corresponds to AND logic (Figure 14c). ii) When *V*_DS_ is large enough, WS_2_ shows an ambipolar transfer curve, Schottky barrier between the drain and channel will be thinner. When both inputs are set to logic 0 (−6 V), two barriers bend upward, only holes are allowed to flow; when both inputs are set to logic 1 (+4 V), two barriers bend downward, and only electrons are allowed to flow. This bias condition corresponds to XNOR logic (Figure 14d). Due to the embedded logic functions of AND and XNOR in one memory cell and the memory function enabled by graphene floating gate, the logic‐memory‐circuits by this transistor can reduce the transistor number from 4 to 1 for AND/NAND gate and with the decreasing number of 8 to 1 for XNOR/XOR logic, which further demonstrates 2D transistor's possibility in further scaling and for future application.

#### 5.3.3 Neuromorphic Computing

As was previously mentioned, the von Neumann bottleneck refers to the difficulties that classical computing has while handling large amounts of data. While a potential solution to this bottleneck is to reduce the physical distance between the processing center and memory units in a single circuit, there is also another option to address this issue, which is inspired by the human brain that has highly parallel computing and adaptive learning capabilities, so‐called the neuromorphic computing. Compared to traditional bulk material, low‐dimensional material with its highly tunable energy band diagram has drawn wide attention in fabricating synaptic devices including memristors, synaptic transistors, optoelectronic synaptic devices, and heterosynaptic devices.^[^ ^144^ ^]^ As the focus of this review, many types of transistors, including floating gate transistors^[^ ^145^ ^]^ and ferroelectric transistors^[^ ^146^ ^]^ have been explored as synaptic devices outside of logic‐in‐memory applications. However, most of these synaptic transistors adopt the planar MOSFET structure, which limits their further scaling in a smaller footprint area. Therefore, in this part a vertical transistor neuron demonstrated by Han et al.^[^ ^147^ ^]^with a footprint area smaller than 4F^2^ will be discussed.

The schematic view of this transistor can be seen in Figure 14e. In this vertical silicon nanowire‐based transistor, an n+ source is located at the bottom of the body and an n^+^ drain at the top, combining the 400 nm diameter of this nanowire, a footprint smaller than 4F^2^ is achieved. The p‐type floating body is isolated by n^+^ source/drain and by gate dielectrics, serving as a quantum well to accommodate carriers. This vertical transistor neuron has integrated excitatory, inhibitory (*E*/*I*) function enabled by gate modulation and myelination function enabled by asymmetric source and drain electrodes. For the E/I function, when V_g_ = −1.5 V is applied (excitatory function, see energy band diagram in Figure 14f), an electron barrier is formed in the channel due to the upward energy band. When a constant I_in_ is applied to the drain electrode, electrons will integrate in the drain and increase the *V*_D_ (*V*_out_) until it reaches latch‐up voltage (*V*_latch_), the single transistor latch (STL) is triggered under this scenario because of the impact ionization induced by the high lateral electric field. Therefore, the integrated charges in the drain can be fired into the channel. When V_g_ = 1 V is applied (inhibitory function), electrons can't integrate into the drain even though Iin is applied. This is because the device is at LRS due to a pre‐existing inversion layer and the charges escape to the body from the drain via the inversion layer. The spiking characteristic clearly shows the performance of this vertical transistor (Figure 14g). Excitatory function (V_g_ = −1.5 V) enables *V*_out_ to gradually change followed by a sudden drop, which resembles the integrate‐and‐fire (LIF) mechanism that the neuron receives excitatory signals from the previous synapse, and an output action voltage is produced when the membrane voltage reaches a threshold. Inhibitory function on the contrary suppresses the neuron spiking, the *V*_out_ will remain consistently low.

As for the myelination function, this vertical transistor has a natural difference in parasitic capacitance at source and drain electrodes due to a large common source and a small‐sized individual drain, which resembles the myelinated/unmyelinated neuron. In a biological nerve system, the membrane capacitance will decrease in a myelinated neuron due to a lipid‐rich layer, an unmyelinated neuron does not have this layer hence the membrane capacitance is larger. As a result, the myelinated neuron produces a higher spiking frequency (*f*) than the unmyelinated neuron, and the spikes transmitted to the connected synapses are generated more frequently. Similarly, when *I*_in_ is applied to the source, *f* becomes smaller (slower) compared with the counter‐case where *I*_in_ is applied to the drain (Figure 14h). The former situation corresponds to an unmyelinated neuron while the latter situation corresponds to a myelinated neuron. Practically speaking, unmyelinated neuron enhances energy efficiency since spiking appears less frequently while myelinated neuron enhances data transmission speed. Combining the intrinsic low power consumption of this transistor (peak power consumption of 3.6 µW and averaged power consumption of 52.2 nW at *I*_in_ of 20 nA), this transistor has the potential to improve upon the low energy efficiency and limited computational power of current artificial neural networks.

#### 5.3.4 Machine Learning

Machine learning has drawn great interest in developing practical software for computer vision, speech recognition, natural language processing, and robot control due to its capability of self‐improvement through experience or massive given data.^[^ ^148^ ^]^ The support vector machine (SVM), a supervised machine learning algorithm, is an efficient classification tool. However, for practical problems, most of the given data in input space are nonlinear and non‐separable, a kernel function is typically employed to map non‐separable inputs from input space into a higher dimensional space, thus making linear non‐separable inputs to be sparable and easier to classify. Gaussian function, as the most commonly used kernel, has already been demonstrated in CMOS,^[^ ^149^ ^]^ and the circuits were studied for hardware implementations of machine learning algorithms.^[^ ^150^ ^]^ However, scalability issues limit the use of CMOS for tunable Gaussian function generation. However, combing the tunability of heterojunctions composing 2D materials is possible to further reduce transistor numbers in generating kernel functions. In the following part, a mixed‐kernel heterojunction (MKH) transistor demonstrated by Yan et al. will be discussed.^[^ ^151^ ^]^

This transistor's schematic view can be seen in Figure 14i. The total transistor is at micron size. Semi‐vertical geometry of this device design allows for the dual gating of both overlapping and non‐overlapping regions of n‐type MoS_2_ and p‐type CNTs in the heterojunction. The overlap region of the MoS_2_/CNT heterostructure forms a p–n junction diode with nanomaterial‐enabled partial electric‐field screening. Combining the dual gates modulation, by changing different gate voltages on the top and bottom gate can realize the Gaussian function, sigmoid function, and mixed‐kernel functions. Figure 14j (left) shows Gaussian kernel functions with a tunable mean (*µ*) yielded by charging back gate voltage. Now by giving randomly generated data with a concentric distribution shape (Figure 14j, middle) and using a Gaussian kernel generated by this transistor to classify the data, the data can then be classified linearly with a hyperplane in the resulting 3D output space (Figure 14j, right). Also, by varying the area of the overlapping region (overlap length of 10 µm), sweeping the top gate (*V*_TG_), and keeping the back gate fixed at 5 V, this transistor can generate optimized sigmoid kernel functions (Figure 14k, left). Similarly, the given data (Figure 14k, middle) can be linearly classified in a higher dimensional space (Figure 14k, right). Additionally, by tuning back gate voltage, a series of transfer characteristics (mixed kernel) can be generated that contain both sigmoid and Gaussian characteristics (Figure 14l). The Author also demonstrated the mixed‐kernel SVM for arrhythmia detection (Figure 14m). The mixed kernel with a *β* value of 1 V yields the highest average arrhythmia detection accuracy with all the six arrhythmia types being detected with accuracy at or above the ≈90% level and is an improvement compared with conventional purely Gaussian (*β* = −5 V) or purely sigmoid (*β* = 3 V) kernels. At last, a comparison between CMOS implementation and MKH classification has been made. CMOS requires >100 devices to generate a similarly complete set of tunable mixed kernels as can be achieved with only two MKH transistors (Figure 14n, left) and the total number of devices required for SVM hardware with personalized kernel functionality in *n* × *n*‐kernel matrix compared with MKH transistors have been plotted (Figure 14n, right), showing a more obvious scaling advantage for the MKH transistor.

## 6 Summary and Outlook

In conclusion, this review has introduced several types of small feature‐size transistors based on low‐dimensional materials, including vertical transistors, nano‐gate transistors, and nanofabrication‐enabled transistors, whose typical performance metrics along with that of Si‐based devices are shown in **Table** **1**. Significantly, transistors based on low dimensional material have already achieved the smallest gate length down to 0.34 nm^[^ ^6^ ^]^ and the smallest channel length down to 0.65 nm,^[^ ^48^ ^]^ which greatly exceed the feature size achieved by the state‐of‐the‐art V‐shaped Si FET^[^ ^6^ ^]^ and the IRDS (International Roadmap for Devices and Systems) requirement for 2037. Such extremely small feature size can be attributed to the atomic level thickness of low dimensional materials and their excellent electrical properties at the atomic scale. For bulk materials (i.e., Si), reducing their thickness to the nanometer or sub‐nanometer range will cause severe structural defects, which will further damage their properties,^[^ ^37^ ^]^ making them unsuitable for further scaling. Besides, low dimensional materials‐based transistors also have optimized performance metrics compared to Si‐based FET due to their extraordinary intrinsic electrical properties. For example, the 0.34 nm‐gate transistor shows comparable DIBL and leakage current with V‐shaped Si FET due to larger effective electron mass and bandgap of MoS_2_, which contributes to low power consumption and very large‐scale integration applications. Moreover, the dangling bond‐free surface of 2D materials endows them with high mobility at the ultrashort gate/channel length. As shown in Table 1, a 9 nm‐channel length transistor fabricated by Tian et al. in 2023^[^ ^127^ ^]^ possesses mobility of 72 cm^2^V^−1^ s^−1^, which is even higher than the IRDS requirements for 2037 (40 cm^2^ V^−1^ s^−1^) with a larger channel length, which means 2D materials can still be sufficient for fabricating the high‐speed device.

**Table 1:** Summary of the small feature‐size transistors.

Reference	*L*_ch_/*L*_g_ [nm]	µ [cm^2^ V^−1^s^−1^]	*R*_c_ [kΩ·µm]	*I*_on_ [µA µm^−1^]	*I*_off_ [pA µm^−1^]	DIBL [V V^−1^]	SS [mV dec^−1^]	*I*_on_/*I*_off_
Vertical transistors								
[47]	10_ch_	/	/	≈10^2^	≈10^2^	/	450	10^6^
[48]	0.65_ch_	/	/	≈10^3^	≈10^9^	/	/	26
[48]	3.6_ch_	/	/	≈40	≈10^4^	/	/	10^3^
[68]	≈7_ch_	/	/	/	/	/	/	10^7^
Nano‐gate transistors								
[71]	1_g_	/	/	≈10	≈10	0.2	65	10^6^
[75]	≈4_g_	50	/	/	/	/	73	10^5^
[77]	≈4.3_g_	/	/	≈10^−3^	≈10^−3^	/	6.1	10^6^
[6]	0.34_g_	/	/	≈0.2	≈1	1	210	10^5^
[95]	100_g_	/	/	/	/	/	>450	10^3^
[97]	140_g_	/	≈0.79	3320	/	/	/	/
Nano‐fabrication based transistors								
[102]	90_g_	/	/	≈10^3^	≈10^4^	0.179	142	10^5^
[109]	10_g_	30	≈2.5	425	/	/	250	/
[112]	30_ch_	/	/	≈533	≈5 × 10^5^	/	105	≈10^3^
[116]	90_ch_	/	/	≈0.5	≈5 × 10^3^	/	/	10^2^
[120]	70_ch_	/	/	/	/	/	/	10^7^
[122]	3.8_ch_	30	3.8	≈9	≈0.3	0.425	93	10^7^
[123]	7.5_ch_	17.4	10.4	45	≈8	0.14	120	10^7^
[124]	8.2_ch_	1.1	/	2.5	3	/	140	10^6^
[125]	30_ch_	/	/	≈10^3^	≈10^2^	/	83	10^4^
[127]	9_ch_	72	/	433	≈10	50	100	10^7^
IRDS for 2037								
[*]	12_g_	40	/	547	/	/	65	/
V‐shaped Si FET								
[6]	3_g_	/	/	∼10^3^	∼1	0.96	188	∼10^9^

* Number_g_ and Number_ch_ means the gate length and channel length, respectively.

* Data from the website: https://irds.ieee.org/editions

Although these small‐sized devices outperform silicon in certain performance metrics, they still encounter different challenges due to differences in structure and fabrication techniques. For vertical transistors, it is easy for them to achieve smaller channel lengths even compared to other transistors in this review due to the natural atomic thickness of 2D materials and their compatibility with vdW integration. In addition, vdW heterojunctions are beneficial for alleviating the Fermi pinning effect, which is also helpful in enhancing barrier height modulation by V_g_ and further scaling vertical channel length. Nevertheless, a trade‐off must be made between the on‐state current (device speed) and on–off ratios (power consumption) when the channel length is scaled down toward a few atomic‐layer thicknesses (We have listed two transistors from^[^ ^48^ ^]^ with different channel lengths in table) due to suppressed thermionic emission and enhanced tunneling current of an enlarged bandgap of the channel material,^[^ ^48^ ^]^ a possible optimizing solution is to use thickness‐independent bandgap TMD such as ReS_2_. Besides, since thin film graphene electrodes in vertical transistors still have a weak screening effect on back gate modulation, a porous bottom electrode with a proper Schottky barrier under the channel can help increase gate modulation, alleviating severe SCEs in the ultrashort vertical channel.^[^ ^48^ ^]^ For nano‐gate transistors with 1D CNTs or NWs as gates, they have a natural advantage in scaling gate length due to ultrasmall gate diameter. However, most nano‐gate transistors discussed here have a common drawback: the physical length between drain/source electrodes is much larger than gate length (even three orders of magnitude larger in^[^ ^75^ ^]^), resulting in a weakened gate field effect. To solve this issue, alternative methods, such as heterophase engineering and nanogap fabrication, have been introduced to make nano‐gate transistors further achieve a small footprint. Additionally, the self‐aligned fabrication method put forward by Liao et al. is also an effective way to improve gate modulation and decrease access resistance by decreasing effective channel length, though the physical length between two external electrodes remains large. For nanofabrication‐based devices, the channel length of most small feature‐size transistors may be limited by the resolution of EBL, while new methods were proposed to fabricate nanogap under 10 nm, namely directly determining nanogap between electrodes or building nanogap dielectric. Such methods may be suitable to fabricate wafer‐scale nanogap transistor arrays. However, larger off‐currents could be observed in such transistors due to the enhanced DIBL^[^ ^127^ ^]^ or the influence of the limited quality of nanogap dielectric during the deposition process.^[^ ^125^ ^]^

By comparing the advantages and limitations of these types of transistors, it can be inferred that small feature‐size transistors based on low‐dimensional materials are of great significance for the development of highly integrated transistor devices, but further development and research are also needed. Therefore, we have put forward prospects for their future development as shown below.

### 6.1 Performance Optimization for Small‐Size Transistors

Although 2D materials offer potential solutions to mitigate SCEs when compared to silicon, thanks to their larger bandgap, heavier effective electron gas, and lower in‐plane dielectric constant, there are still additional challenges to address. One such challenge is optimizing the contact resistance between the 2D material and metal electrode,^[^ ^152^ ^]^ which arises from the Schottky barrier. This can be achieved through direct modulation of the Schottky barrier or by identifying metals with similar work functions to form Ohmic contacts. Alternatively, researchers are exploring novel methods to overcome this issue. Achieving smaller SS is also an important issue in achieving lower energy consumption. However, the scaling of the supply voltage in the ICs cannot keep up with the shrinking of the characteristic size of conventional transistors. To be specific, a minimum 60 mV gate voltage is needed to magnify drain current by one order at room temperature. Therefore, developing new transistor structures^[^ ^153^ ^]^ or using ferroelectric dielectric^[^ ^154^ ^]^ is an alternative way to break 60 mV limits. Nevertheless, more works are needed to successfully apply these transistor structures in ultrasmall footprint scale and further figure out capacitance matching between ferroelectric capacitance and depletion region capacitance in short channel conditions. What's more, since a high‐electric field and defective interface between channel and gate oxides induced electron mobility degradation is almost inevitable when the channel is extremely shortened and thinned, it is important to find ways to alleviate this degradation. Until now, by adopting a fewer‐defects transfer method, vdW gap enlargement^[^ ^155^ ^]^ and rapid pulsing (fast switching speeds)^[^ ^156^, ^157^ ^]^ are found to be useful for achieving a clean interface/reaching a high saturating velocity.

### 6.2 Large‐Scale Array Fabrication of Ultrasmall Transistors

Large‐scale array production presents issues for small‐footprint transistors, particularly for those ultrasmall transistors. For instance, while a mechanical break‐induced nanogap transistor can effectively control the size of the gap (or channel) to as low as 1 nm by bending the substrate, this approach becomes highly impractical for large‐scale fabrication when multiple transistors are produced on a single substrate. Similarly, electron beam lithography, though capable of fabricating ultrasmall transistors, is hindered by its excessively time‐consuming nature, posing challenges for large‐scale applications. Therefore, it is important to achieve scalable fabrication for ultrasmall transistors in order to apply them in future ICs. Until now, there are some methods that have been developed for fabricating small transistors on a large scale,^[^ ^158^, ^159^, ^160^ ^]^ and research into the production of a large‐scale array of ultrasmall transistors must continue.

### 6.3 Exploration of New Device Structure

Despite all these transistors we have mentioned above, some other advanced structures, such as sloping‐channel transistors^[^ ^161^ ^]^ and multidirectional channel transistors^[^ ^162^ ^]^ have the potential to further continue Moore's law. Designing other kinds of devices instead of focusing on the transistor's configuration is also an alternative way. The greater number of integrated circuits corresponds to a higher quantity of transistors, as highlighted in the introduction. This, in turn, can result in enhanced processing capacity, but it also entails an increase in power consumption. Not to mention that the scaling is also facing its limitations. For example, a 0.34 nm graphene gate (put forward by Ren et al.) is believed to be the smallest size to form a controlling gate up to now,^[^ ^163^ ^]^ which puts an ultimate limitation for further scaling the gate length unless new materials can be found. What's more, higher consumption is undesirable but also inevitable for achieving better computing power. Now, some research—such as neuromorphic devices (or, brain‐like devices)—may have undermined the conventional device architecture and current operating principle. As a result, there is significant potential for enhancing performance beyond transistor‐based circuits and eliminating the von Neumann bottleneck at the device level through the utilization of electronic devices that emulate the human brain.^[^ ^164^, ^165^, ^166^ ^]^

## Conflict of Interest

The authors declare no conflict of interest.
